# Comparative analysis of C‐type lectin domain proteins in the ghost moth, *Thitarodes xiaojinensis* (Lepidoptera: Hepialidae)

**DOI:** 10.1111/1744-7917.12564

**Published:** 2018-02-22

**Authors:** Qian Meng, Ji‐Hong Zhang, Huan Zhang, Gui‐Ling Zhou, Ruo‐Yao Ni, Yan‐Ni Zhao, Qi‐Lian Qin, Zhen Zou

**Affiliations:** ^1^ State Key Laboratory of Integrated Management of Pest Insects and Rodents, Institute of Zoology Chinese Academy of Sciences Beijing China; ^2^ College of Life Sciences Hebei University Baoding Hebei China; ^3^ University of Chinese Academy of Sciences Beijing China

**Keywords:** binding ligand, carbohydrate‐recognition domain, C‐type lectin domain protein, expression profiling, phylogenetic feature, *Thitarodes xiaojinensis*

## Abstract

Insects have a large family of C‐type lectins involved in cell adhesion, pathogen recognition and activation of immune responses. In this study, 32 transcripts encoding C‐type lectin domain proteins (CTLDPs) were identified from the *Thitarodes xiaojinensis* transcriptome. According to their domain structures, six CTLDPs with one carbohydrate‐recognition domain (CRD) were classified into the CTL‐S subfamily. The other 23 CTLDPs with two CRDs were grouped into the immulectin (IML) subfamily. The remaining three with extra regulatory domains were sorted into the CTL‐X subfamily. Phylogenetic analysis showed that CTL‐S and CTL‐X members from different insects could form orthologous groups. In contrast, no *T. xiaojinensis* IML orthologues were found in other insects. Remarkable lineage‐specific expansion in this subfamily was observed reflecting that these CTLDPs, as important receptors, have evolved diversified members in response to a variety of microbes. Prediction of binding ligands revealed that *T. xiaojinensis*, a cold‐adapted species, conserved the ability of CRDs to combine with Ca^2+^ to keep its receptors from freezing. Comparative analysis of induction of CTLDP genes after different immune challenges indicated that IMLs might play critical roles in immune defenses. This study examined *T. xiaojinensis* CTLDPs and provides a basis for further studies of their characteristics.

## Introduction

Insects live in a hostile environment and possess effective immune systems (Lemaitre & Hoffmann, 2007). The initial step in the initiation of immune responses relies on biosensor proteins called pattern recognition receptors (PRRs), which can detect and bind to certain pathogen‐associated molecular patterns (PAMPs) on the surface of invading microbes. PAMPs consist of lipopolysaccharides (LPS) or peptidoglycans (PGNs) in bacteria and β‐1, 3‐glucans in fungi (Pal & Wu, 2009; Wang *et al*., 2017). Insects have evolved a diverse group of PRRs corresponding to different PAMPs.

C‐type lectins constitute a large family of PRRs that have been identified in many organisms, ranging from plants, animals to viruses (Wang *et al*., 2013). In most cases, binding of the carbohydrate‐recognition domain (CRD) to sugars requires ligation to Ca^2+^. However, evidence shows that not all C‐type lectins have the ability to associate with carbohydrates or Ca^2+^ (Zelensky & Gready, 2005). We therefore employed annotation studies in *Manduca sexta* and *Bombyx mori* to use C‐type lectin domain proteins (CTLDPs) to refer to proteins harboring the domains (Rao *et al*., 2015a, b).

The genomes of many arthropods have been sequenced and a large number of CTLDPs have been identified. There are 34, 25, 39, 12, 16, 24, 34 and 23 genes encoding CTLDPs identified in the model insect species, *Drosophila melanogaster*, *Anopheles gambiae*, *Aedes aegypti*, *Apis mellifera*, *Tribolium castaneum*, *Helicoverpa armigera*, *M. sexta* and *B. mori*, respectively (Evans *et al*., 2006; Waterhouse *et al*., 2007; Zou *et al*., 2007; Xiong *et al*., 2015). Each CTLDP from the Dipteran and Hymenopteran species as listed contains a single CTLD and many of them are involved in immune responses (Table S1). For instance, *D. melanogaster* DL1, 2 and 3 with galactose specificity can agglutinate bacteria, and the latter two enhance melanization and activation of prophenoloxidase (PPO) (Tanji *et al*., 2006; Ao *et al*., 2007). AgCTL4 and AgCTLMA2 in the mosquito *A. gambiae* prevent ookinetes of *Plasmodium berghei* from melanization (Osta *et al*., 2004). A novel CTLDP from *A. aegypti* (CLSP2), composed of elastase‐like serine protein at the N‐terminus and CTLD at the C‐terminus, was shown to be a negative regulator in anti‐*Beauveria bassiana* infection (Wang *et al*., 2015). In Lepidoptera, the number of CTLDs in CTLDPs is distinctive. Besides the proteins present in one CTLD, there are also extraordinary CTLDPs possessing dual and triple CTLDs (Rao *et al*., 2015a, b). CTLDPs with two CTLDs are also found in *T. castaneum* (Zou *et al*., 2007). To date, Lepidoptera CTLDPs involved in immune responses were mainly found in proteins with two tandem CRDs (Table S2). For example, *M. sexta* immulectins (MsIML1–4) can be induced by bacteria and fungi, stimulating agglutination of the bacterial and fungal cells (MsIML1 and 4) and moderating PPO activation (MsIML1, 2 and 4) (Yu *et al*., 1999, 2005, 2006). In *B. mori*, the immune functions of IML1, 3, 4 and 5 have been investigated (Koizumi *et al*., 1999; Watanabe *et al*., 2006; Takase *et al*., 2009). In crustaceans, CTLDPs have also been identified and reported to be involved in immune defenses. For instance, a *Pacifastacus leniusculus* CTLDP with a single CRD plays a regulated role in prophenoloxidase activation (Wu *et al*., 2013).

*Thitarodes xiaojinensis* is a major host of *Ophiocordyceps sisnensis* (Zhang & Tu, 2015). Previously, 258 immunity‐related genes were identified in the transcriptome of *T. xiaojinensis* (Meng *et al*., 2015). The genus *Hepialus* is a phylogenetically primitive group of Lepidoptera (Nielsen *et al*., 2000). Therefore, analysis of the phylogenetic relationship between CTLDPs in *T. xiaojinensis* and those in other insects will help us understand if they share a common ancestor or emerged individually after divergence of phyla. To address this goal, transcriptome data were searched and we used recently released information on the CTLDPs of *M. sexta* and *B. mori*. A total of 32 transcripts encoding CTLDPs were annotated. These CTLDPs were classified into three groups based on domain structure. The lineage‐specific expansion in *T. xiaojinensis* CTLDPs is associated with expansion of proteins with tandem CTLDs. Additionally, expression profiling of the CTLDP genes after different immune challenges was used to explore their roles in *T. xiaojinensis* immunity.

## Materials and methods

### Identification and characteristic prediction of genes encoding CTLDPs in T. xiaojinensis

C‐type lectin domain protein amino acid sequences from *M. sexta*, *B. mori* and *A. aegypti* (Shin *et al*., 2011; Rao *et al*., 2015a, b; Wang *et al*., 2015) were accessed as queries to search the *T. xiaojinensis* unigene database (Meng *et al*., 2015). In total, 32 transcripts were confirmed by sequencing and deposited in the GenBank database (Table S3). The functional domain was detected using SMART (http://smart.embl.de/). Their signal peptides and transmembrane regions were predicted through CBS prediction server (http://www.cbs.dtu.dk/services/). Their domain architectures were visualized by software DOC (Ren *et al*., 2009).

### Sequence alignments and analysis of phylogenesis

Subfamily‐specific CTLDPs from other insects were retrieved through BLASTP. Those sequences along with corresponding *T. xiaojinensis* CTLDPs were aligned by CLUSTALX 2.1 with the following settings: weight matrices of BLOSUM series, a gap penalty of 10 and an extension gap penalty of 0.1 (Thompson *et al*., 1997). According to alignment results, MEGA 6.0 was implemented to build unrooted neighbor‐joining trees using the bootstrap method (Tamura *et al*., 2013).

### Prediction for ligand binding site and three‐dimensional (3D) model

Protein‐ligand binding sites in *T. xiaojinensis* CTLDs were predicted by the COACH (http://zhanglab.ccmb.med.umich.edu/COACH/) along with the I‐TASSER server (Roy *et al*., 2010; Yang *et al*., 2013) and denoted manually in the alignment of CTLDs. Putative 3D models were generated by I‐TASSER and shown using the PyMOL molecular graphic system.

### Induction analysis of T. xiaojinensis CTLDPs after different microbial challenges

To investigate changes in transcriptional levels after immune stimulation, 10 complementary DNA (cDNA) libraries, referring to messenger RNA (mRNA) samples from the fat body of larvae without treatment (control group) and larvae exposed with *Enterobacter cloacae* for 6 h, Ringer's solution (8.05 g NaCl, 0.42 g KCl and 0.18 g CaCl_2_ per L) for 12 h, *Ophiocordyceps sinensis* for 12, 48, 72 h, 1 year, and *Cordyceps militaris* for 12, 48, 72 h, were constructed and separately sequenced on the Hiseq 2000 (Meng *et al*., 2015). The fragments per kilobase per million mapped reads (FPKM, representing transcript abundance) value of each gene encoding CTLDP was calculated and used to determine fold change by the DEGseq package in R https://www.r-project.org/about.html (*P*‐value < 0.001). Following this, values of log_2_‐fold change for 32 *T. xiaojinensis* CTLDP genes were used to draw a heatmap using heatmap.2 function in R environment. To validate the results of differentially expressed genes (DEGs), specific primers (Table S4) for six genes were designed to carry out quantitative real‐time polymerase chain reaction (qPCR). The cDNAs from the 10 libraries were used as the templates. The detailed procedure has been previously described (Meng *et al*., 2015). The acquired data were exported to Excel for computing ‐ΔΔCt, corresponding to log_2_‐fold change exhibiting in the heatmap.

## Results

### Features of T. xiaojinensis CTLDPs

**Figure 1:**
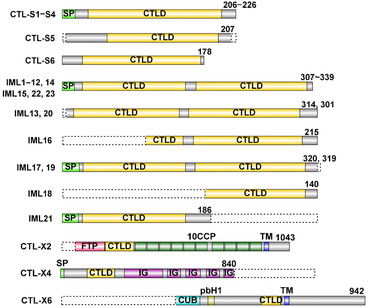
Schematic architectures of the 32 *Thitarodes xiaojinensis* C‐type lectin domain proteins (CTLDPs). The putative signal peptide (SP), CTLD, eel‐fucolectin tachylectin‐4 pentaxrin‐1 (FTP), complement control protein (CCP) (also known as sushi), immunoglobulin (Ig), complement C1r/C1s, Uegf, Bmp1 (CUB), parallel beta‐helix 1 (pbH1) and transmembrane domains (TM) are in different colored boxes. The sizes or size ranges of CTLDPs are indicated on the top right of each bar. The dashed boxes in the N‐ or C‐terminal ends represent the missing regions. The size of individual architectures is not proportional.

There are 32 CTLDPs identified in the *T. xiaojinensis* transcriptome (Table S3) and these were divided into three subfamilies. Six proteins presenting a single CTLD were assigned to the CTL‐S subfamily, while 23 proteins containing two CTLDs were grouped into the immulectin (IML) subfamily; the remaining three proteins have more complex structures including both CTLDs and other conserved domains (Fig. 1). These were assigned to the CTL‐X subfamily. Initiation and/or stop codons could not be found in the coding sequences of CTL‐S5, IML13, 16–21, CTL‐X2, X4 and X6 (Table S3); therefore, the N‐ or/and C‐terminal regions in their architectures, as shown in Figure 1, were absent. Previous studies indicated that CTLs, possessing a signal peptide but not a transmembrane domain, execute their functions extracellularly (Table S1 and S2), and thus *T. xiaojinensis* CTL‐S1–S4, IML1–12, 14, 15, 17, 19, 21–23 and CTL‐X4 probably similarly work. Functions of CTLs with a transmembrane region were reported more in vertebrates. These receptors anchor to cell membrane and generally interact with ligands by their extracellular parts (Zelensky & Gready, 2005). *T. xiaojinensis* CTL‐X2 and X6 containing transmembrane regions are expected to work in a similar way. CTL‐S6 without the N‐terminal secretion signal or C‐terminal transmembrane region may be retained in the cytoplasma.

CTL‐S1, S2 and S3 contain the QPD motif, which may bind galactose (Table S3). In contrast, the CRD in CTL‐S6 is likely to interact with mannose, as it contained the EPN motif. The carbohydrate‐recognition motif in CTL‐S4 and CTL‐S5 presents an untypical signature (QRT and HPL, respectively), and thus their characteristics need further investigation. The EPN motifs are mostly localized in the first CRDs of *T. xiaojinensis* IML, namely IML1A, 2A, 4A, 7A, 8A, 10A, 15A–17A, 23A, while the QPD motifs are mostly localized in the second CRDs of *T. xiaojinensis* IML, namely IML1B, 3B–5B, 7B–11B, 13B–15B, 17B, 22B, 23B (Table S3). The two CRDs in IML6 are the same, namely EPN motifs, while the dual CTLDs in IML20 present two QPD motifs. IML12 and IML19 are exceptional; the motifs in the two CTLDs of the former are QPN and RRN; the motif in the first CRD of the latter is QPD (Table S3).We identified three proteins in *T. xiaojinensis* belonging to the CTL‐X group (Table S3). The conserved carbohydrate‐recognition motif, QPD, was only found in the CRD of *T. xiaojinensis* CTL‐X2 (Table S3). The CRD in CTL‐X4 contains a non‐canonical motif (DNH) and the motif for binding carbohydrate could not be identified in CTL‐X6 (Table S3). Even so, they might be involved in cell–cell interactions or complement activation due to the immunoglobulin (Ig) (in CTL‐X4) and CUB (complement C1r/C1s, Uegf, Bmp1) (in CTL‐X6) domain.

### Evolutionary relationships among insect CTLDPs

**Figure 2:**
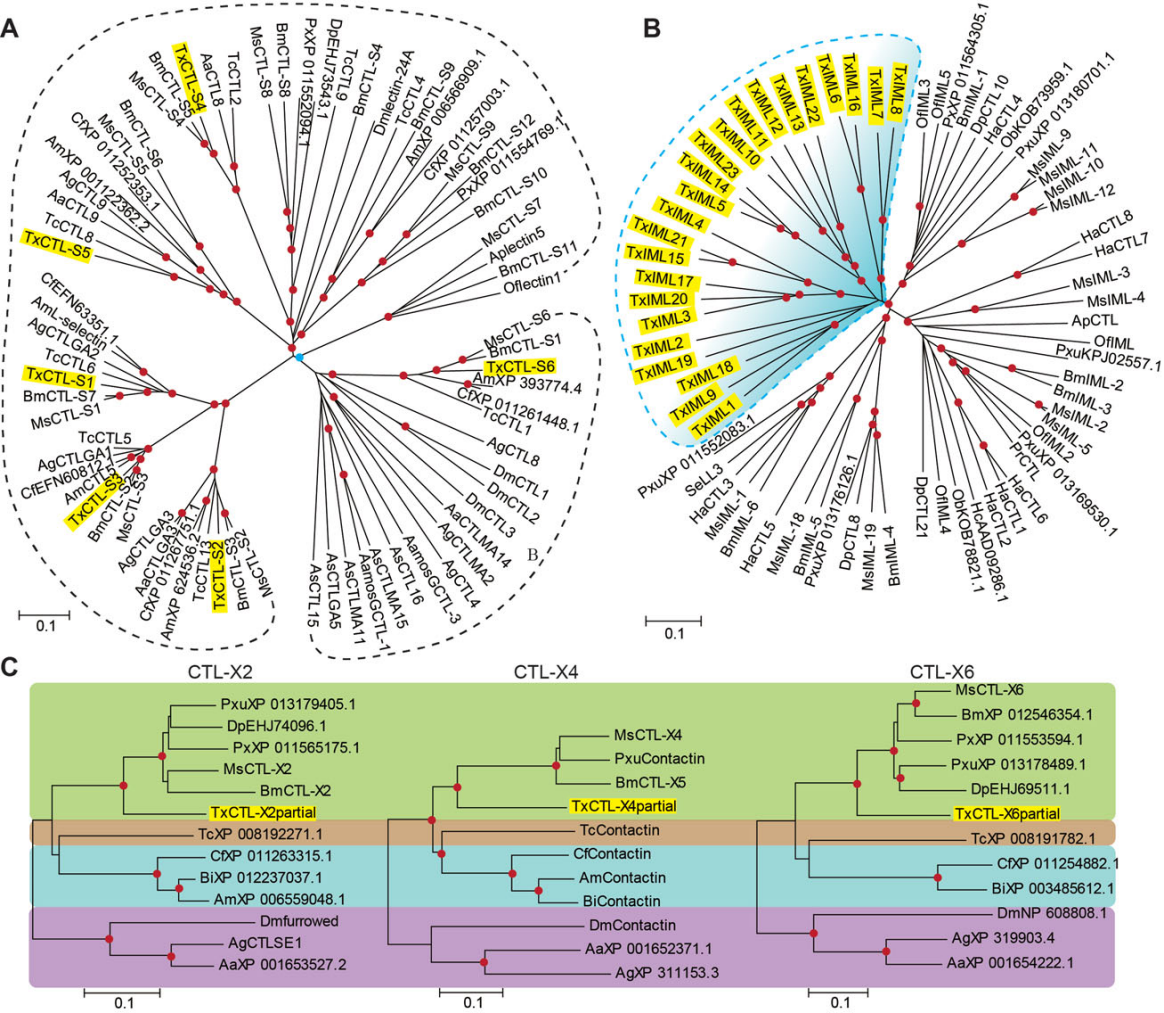
Phylogenetic relationships between C‐type lectin domain proteins (CTLDPs) of *Thitarodes xiaojinensis* (shaded yellow) and other insect species. Members belonging to *T. xiaojinensis* CTL‐S, immulectin (IML) and CTL‐X were separately analyzed with their orthologues, which present similar domain structures. (A) CTL‐S family. The evolutionary tree was separated into two clusters. Bootstrap value of the blue node is 963 (1000 trials). (B) IML family. Lineage‐specific expansion of *T. xiaojinensis* IMLs is denoted by blue shade. (C) CTL‐X family. Four divided subgroups are exhibited in all three CTL‐X trees and shaded in different colors. Each of green, pink, blue and purple clades only include Lepidoptera, Coleoptera, Diptera and Hymenoptera, respectively. Red dots at the nodes indicate bootstrap values >700 from 1000 trials. Amino acid sequences without names were represented by their Genbank accession numbers. Tx, *T. xiaojinensis*; Ms, *Manduca sexta*; Bm, *Bombyx mori*; Px, *Plutella xylostella*; Dp, *Danaus plexippus*; Of, *Ostrinia furnacalis*; Se, *Spodoptera exigua*; Pxu, *Papilio xuthus*; Ob, *Operophtera brumata*; Hc, *Hyphantria cunea*; Pr, *Pieris rapae*; Ap, *Antheraea pernyi*; Tc, *Tribolium castaneum*; Am, *Apis mellifera*; Cf, *Camponotus floridanus*; Dm, *Drosophila melanogaster*; Ag, *Anopheles gambiae*; Aa, *Aedes aegypti*; As, *Armigeres subalbatus*.

Due to the remarkable differences in structures among the three CTLDP subfamilies, the phylogenetic relationships of CTLDPs were separately analyzed. CTL‐S subfamily members were divided into two clades (Fig. 2A). *T. xiaojinensis* CTL‐S1 through S5 formed clade A and might be closely related according to high bootstrap values and CTL‐S6 is located in clade B (Fig. 2A). In addition, *T. xiaojinensis* CTL‐S1 through S6 reveal monophyletic groups with their orthologues from other insects. In *M. sexta* and *B. mori*, three IMLs possess their own 1 : 1 orthologues (Rao *et al*., 2015a, b). However, orthologous pairs of all identified *T. xiaojinensis* IMLs could not be detected. A total of 23 *T. xiaojinensis* IMLs clustered together showing a clear species‐specific expansion (Fig. 2B). *T. xiaojinensis* IML1, 9, 18, IML3, 4, 15, 17, 20, 21, IML5, 10–14, 22, 23 and IML 6–8, 16, exhibited high bootstrap values among each other, and represent four groups of related IMLs that may have evolved through multiple gene duplications.

All three protein sequences of *T. xiaojinensis* CTL‐X were incomplete, but their CTLDs could be detected (Table S3). Considering that the domain architectures among CTL‐X2, X4 and X6 are variable, CTL‐X protein sequences from different insects were individually classified into CTL‐X2, X4 or X6 groups at first, and then members in the same classification were aligned with each other. The three CTL‐X groups revealed similar evolutionary relationships with those in CTL‐Ss. The CTL‐Xs formed strong monophyletic groups (Fig. 2C) suggesting a common ancestor before the divergence of these phyla.

### Phylogenetic and structural characteristics of CTLDs

**Figure 3:**
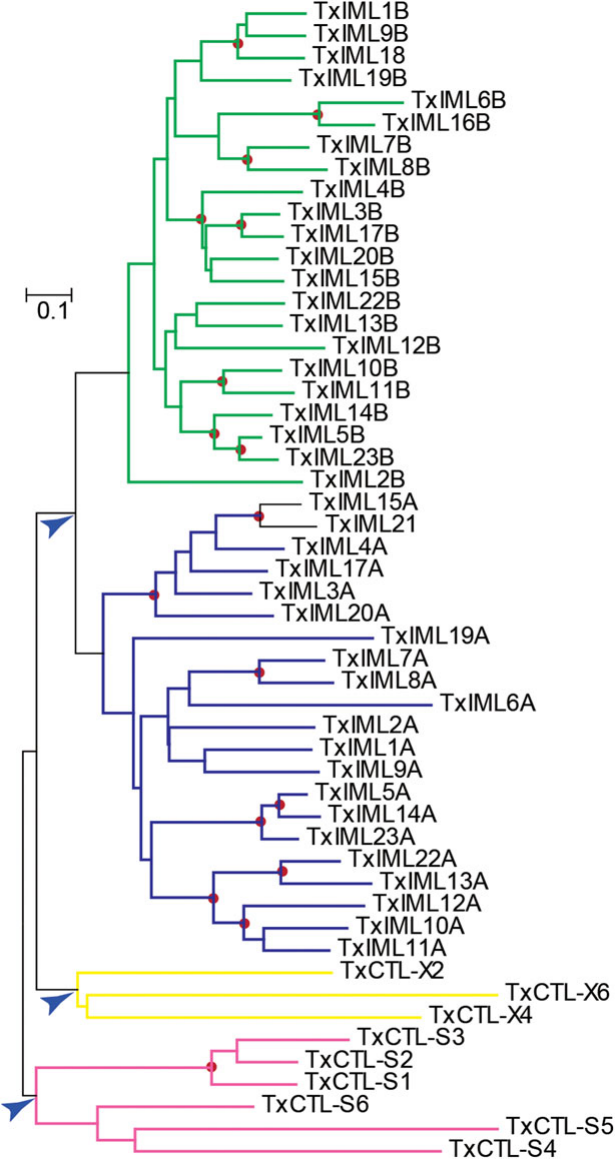
Evolutionary relationships of *Thitarodes xiaojinensis* C‐type lectin domains (CTLDs). A neighbor‐joining tree was built based on the alignment of CTLD sequences. CTLDs from the category of CTL‐S, CTL‐X, immulectin (IML)‐A and IML‐B formed four individual clades shaded pink, yellow, blue and green, respectively. Red dots at the nodes indicate bootstrap values >700 from 1000 trials. Blue arrows denote the key nodes at which the evolution has occurred. There were too few amino acid residues in the IML16A to be included.

Research on the CTLDPs from *T. xiaojinensis* will aid understanding of the phylogenetic relationships among the Hepialidae. To achieve this goal, a neighbor‐joining tree was built based on sequence alignment of 53 *T. xiaojinensis* CTLDs and four clades were revealed (Fig. 3). All the IML‐As (the CTLDs near the N‐terminus) along with IML21 clustered into the blue clade and all the IML‐Bs (the CTLDs near C‐terminus) along with IML18 clustered into the green clade (Fig. 3). The pink clade contained members belonging to CTL‐S, while the yellow clade contained CTL‐X members (Fig. 3). These clustering relationships suggest that CTLDs from the same category evolved from a common ancestor, possessing similar composition of amino acid sequences, and were thus grouped together. The close relationship between IML‐A and IML‐B indicated that gene duplication probably gave rise to the emergence of the ancestor IML genes. The differentiated nodes denoted by blue arrows in Figure 3 suggest that the evolution of the CTL‐S group occurred before the variation pattern of the IML and CTL‐X groups. In addition, phylogenetic relationships of IML‐A members mostly corresponded to those in IML‐B and were also reflected in the IML evolutionary tree (Fig. 2B) derived from complete sequence alignments (i.e. IML3, 4, 15, 17, 20, IML5, 10–14, 22, 23 and IML1, 9, 18).

**Figure 4:**
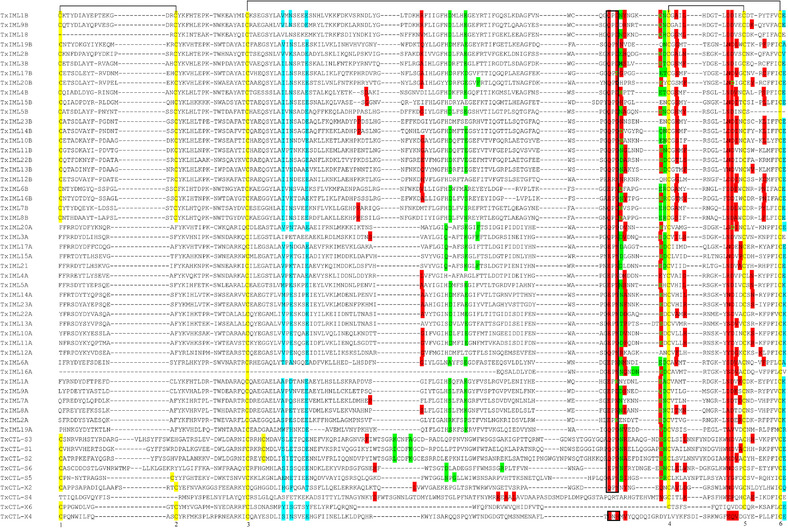
Multiple sequence alignments of C‐type lectin domains (CTLDs) in *Thitarodes xiaojinensis*. The conserved cysteine (Cys) residues are highlighted in yellow and marked with 1 to 6. The predicted disulfide linkages between Cys‐1 and ‐2, ‐3 and ‐6, ‐4 and ‐5 are shown by lines. The motifs (e.g. EPN and QPD) that usually participate in carbohydrate recognition in CTLDPs are indicated by the enclosed box. Residues involving in binding Ca^2+^ in site‐1 and site‐4 are shaded in green and cyan, respectively. Ligand binding sites in each CTLD were predicted through COACH combining with I‐TASSER server. The consensus binding residues are shaded in red, with those also ligating to Ca^2+^ in bold and green font.

To determine whether CTLDs from the same category have common features, the alignment of the 53 CTLD amino acid sequences was further analyzed. Six cysteine (Cys) residues, which may form three disulfide bridges for stability, are conserved in nearly all of the CTLDs belonging to the IML‐A group (Fig. 4). The disulfide linkages in CTL‐S and CTL‐X groups were variable. In the CTL‐S group, Cys‐3 and ‐6 as well as Cys‐4 and ‐5 are thought to build bridges in all members, while Cys‐2 is shifted in CTL‐S1–S3 resulting in its linkage with Cys‐1 being different from the others. All six Cys residues in conserved locations were identified in the CTLDs of CTL‐S5. In the CTL‐X group, CTL‐X2 and X6 maintain the three disulfide bridges in the conserved position. The residues around Cys‐4 revealed category‐specific features. All members in the IML‐B category contained a Gly residue at the first site after Cys‐4, while in members belonging to the IML‐A and CTL‐S categories, the residues at the same position were valine/isoleucine/leucine/alanine (Val/Ile/Leu/Ala).

**Figure 5:**
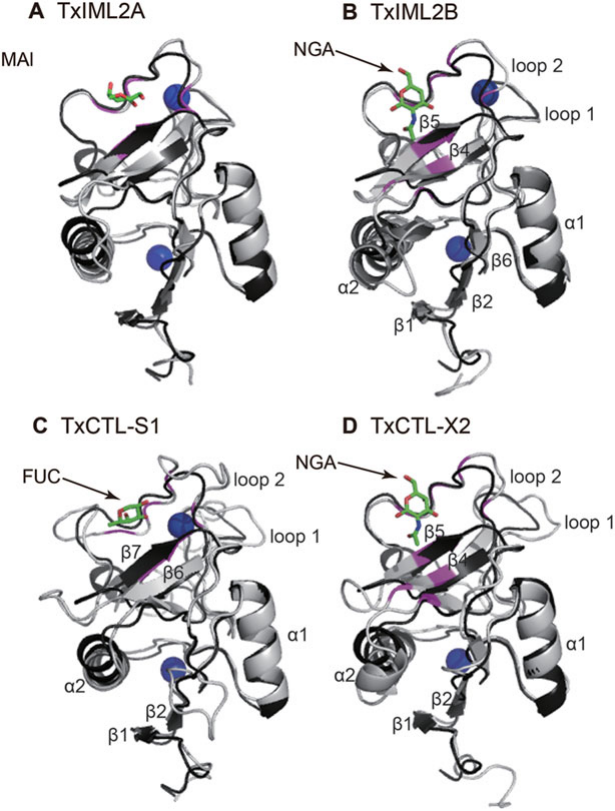
Structures of the C‐type lectin domains (CTLDs) in *Thitarodes xiaojinensis* immulectin (IML)2A (A), IML2B (B), CTL‐S1 (C) and CTL‐X2 (D) and structural comparison with the CTLD in human human dendritic cell‐specific intercellular adhesion molecule‐grabbing nonintegrin‐related (DC‐SIGNR) (1K9J). *T. xiaojinensis* and human CTLDs are colored gray and black, respectively. Secondary structure elements in Figure S1 are labeled in the corresponding models. The blue spheres indicate Ca^2+^ ions. The stick models represent the carbohydrate ligands (the C atoms are shaded in green and the O atoms are in pink). The residues involving in binding carbohydrate (Table S5) are highlighted in magenta. MAN, alpha‐D‐mannose; NGA, N‐acetyl‐D‐glucosamine; FUC, alpha‐L‐fucopyranose.

To predict the functions of *T. xiaojinensis* CTLDPs, potential binding sites and corresponding ligands in the 53 CTLDs were detected by COACH server combining with I‐TASSER (Roy *et al*., 2010; Yang *et al*., 2013). In some CTLDs the residues (in bold) responsible for interaction with carbohydrates were presumed to associate with Ca^2+^ (Table S5). In particular, CTL‐S1–S3, S5, S6, IML1A–3A, 5A, 7A–11A, 13A–17A, 19A, 20A, 21, 22A, 23A, IML1B–11B, 13B, 14B, 16B, 17B, 18, IML20B and 22B might bind sugar ligands in a calcium‐dependent pattern. IML4A, 6A, 12A, 12B, 15B, 19B, 23B, CTL‐X2 and X4 were predicted to combine sugar as well as Ca^2+^, but their binding sites are not shared. Among the remaining CTLDs, one probably binds carbohydrates in the absence of Ca^2+^ and the other might only interact with Ca^2+^ (Table S5). CTLDs possessing EPN motifs usually have characteristics of mannose‐binding and those having QPD motifs usually reveal characteristics of galactose‐binding (van Vliet *et al*., 2008). In *T. xiaojinensis*, IML1A, 2A, 7A, 15A, 17A and 21 containing EPN motifs are likely to combine with mannose, as the predicted results are consistent with the rule. IML19A, 20A, 1B, 3B, 5B, 11B, 22B and CTL‐X2 probably have the ability to bind galactose, since their putative ligands are sugar derivatives of galactose (Table S5). In addition, secondary and tertiary structures of four CTLDs separately from IML‐A, IML‐B, CTL‐S and CTL‐X groups were compared with human dendritic cell‐specific intercellular adhesion molecule‐grabbing nonintegrin‐related (DC‐SIGNR) (1K9J), which is known to recognize mannose type ligands (Probert *et al*., 2013), to determine what properties in the structures determine their binding specificity (Figs. 5 and S1). The four representative *T. xiaojinensis* CTLDs (gray) had a typical CTLD structure model presenting double loops similar to those in DC‐SIGNR (black) (Fig. 5). The 2D structures of CTLDs generally have two β‐sheets at the N‐terminus followed by two α‐helices and two or three β‐sheets at the C‐terminus (Fig. S1). The N‐ and C‐terminal β stands come together to form an antiparallel β‐sheet located at the bottom of the putative crystal structures and the other two C‐terminal β strands (such as *T. xiaojinensis* IML2A β4 and β5) form the second β‐sheet located at the top of the structures (Fig. 5 and S1). The second loop is the primary region participating in binding carbohydrates and/or interaction with Ca^2+^ (Fig. 5). *T. xiaojinensis* IML2A, possesses the EPN motif and is predicted to bind mannose requiring Ca^2+^ coordination (Table S5). Its 3D structure aligned well with DC‐SIGNR (1K9J) (Fig. 5A), especially the region involved with mannose. However, for the other three CTLDPs, the areas involved in sugar binding in the second loops did not match those in DC‐SIGNR. These results were consistent with the prediction in Table S5 that the motifs in *T. xiaojinensis* IML2B, CTL‐S1 and CTL‐X2 are not EPN and their sugar ligands are N‐acetyl‐D‐glucosamine (NGA), alpha‐L‐fucopyranose and NGA, respectively. It indicates that the second loop is structurally flexible among CTLDPs and is the key component of binding specificity. Some *T. xiaojinensis* CTLDs (e.g. CTL‐S2, S3, S6, IML4A, 6A, etc.), had predictions for sugar ligands that were inconsistent with the rule referring to EPN and QPD motifs. Therefore, more evidence will be needed to confirm these predictions.

### Expression profiles of CTLDPs in T. xiaojinensis fat body under various challenges

To investigate mRNA levels of *T. xiaojinensis* CTLDP genes, we searched their FPKM values in ten libraries (Meng *et al*., 2015). The results are listed in Table S6. Overall, genes encoding CTL‐S and CTL‐X expressed at low levels in all conditions. *IML18* and *IML19* presented similar mRNA levels with FPKM values less than 3.5 in untreated/treated groups. High transcript abundances in untreated larvae were observed in genes encoding IML7–9 (Table S6). Except for the five IMLs mentioned above, the remaining IMLs increase their mRNA levels to > 10 or even > 100 after at least one challenge. The maximum value (5768.7) was the *IML1* expression in larvae exposed to *C. militaris* for 48 h (Table S6).

**Figure 6:**
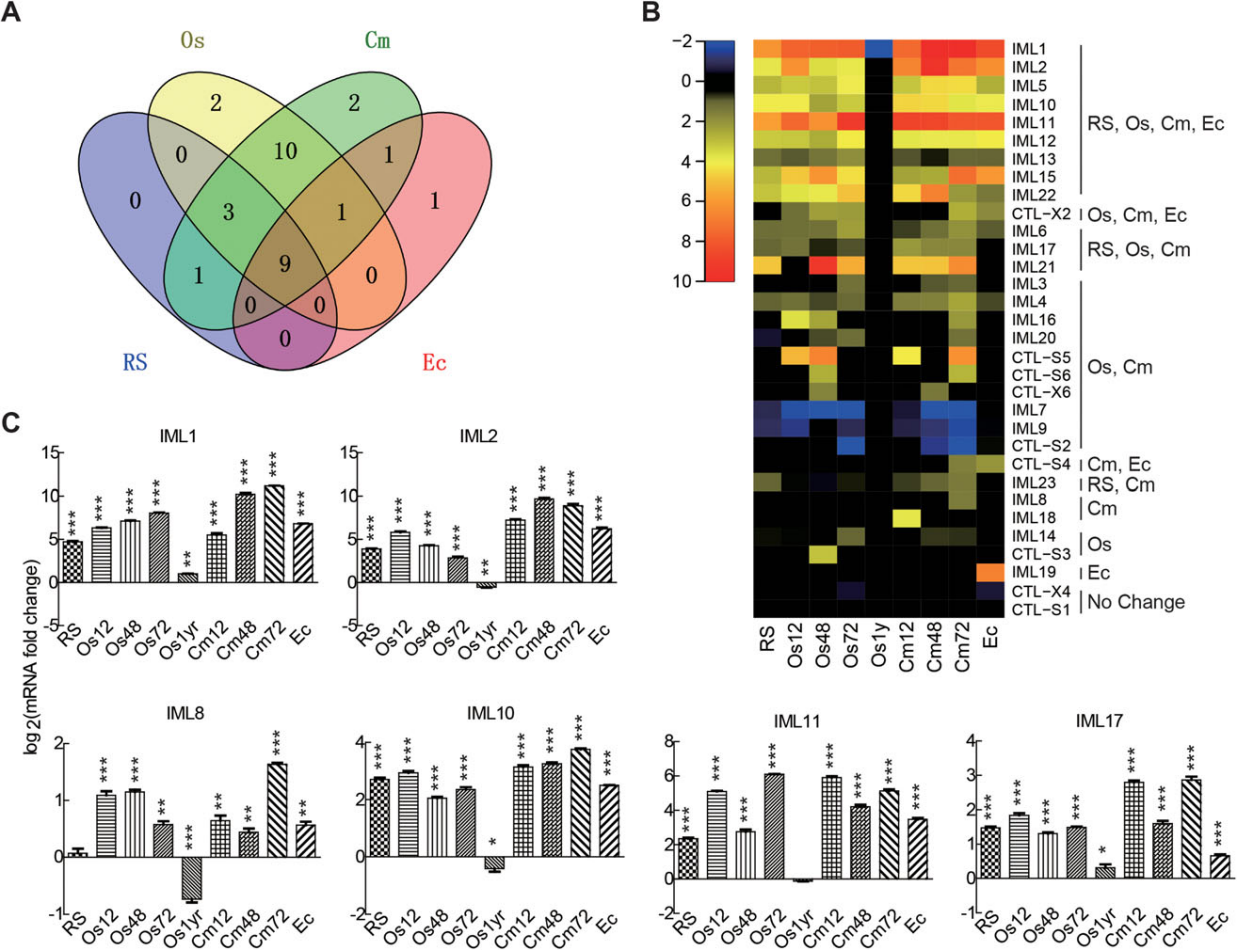
Induced expression of C‐type lectin domain protein (CTLDP) genes in *Thitarodes xiaojinensis* fat body by Ringer's (RS), *Ophiocordyceps sinensis* (Os), *Cordyceps militaris* (Cm) and *Enterobacter cloacae* (Ec) challenges. (A) Venn diagram demonstrating unique or common induction of a gene. The genes shown log_2_‐fold change > 1 or log_2_‐fold change < 0.5 after challenges were considered as differentially expressed genes and submitted to the Venny server (http://bioinfogp.cnb.csic.es/tools/venny/) for statistics. (B) Transcriptional changes of 32 candidate genes after different challenges. Gene boxes were colored based on the value of the log_2_‐fold change. Cm/Os12, Cm/Os48 and Cm/Os72 represent the larvae infected with *C. militaris*/*O. sinensis* for 12, 48 and 72 h. Os1yr represents the larvae infected with *O. sinensis* for a year. (C) Confirmation of differentially expressed genes by quantitative real‐time polymerase chain reaction. *T. xiaojinensis* ribosomal protein S3 (rpS3) was used as an internal control to normalize the templates. Messenger RNA levels are normalized to the control group and expressed as log_2_. Each untreated and treated group contained three larvae. The bars represent the means ± SEM (*n* = 3) from three replications and Student's *t*‐test was used to calculate the statistically significant difference among groups. ^*^ *P* < 0.05; ^**^ *P* < 0.01; ^***^ *P* < 0.001.

To compare induction of *T. xiaojinensis* CTLDP genes after Ringer's, *O. sinensis*, *C. militaris* and *E. cloacae* challenges, DEGs analysis was performed. For fungus *O. sinensis* and *C. militaris* infection, each induced two distinct genes (Fig. 6A). In particular, the mRNA amount of *IML14* individually increased in fat body at 72 h after *O. sinensis* infection, while *CTL‐S3* mRNA levels exhibited 3.1‐fold up‐regulation in the fat body exposed to *O. sinensis* for 48 h; *IML8* and *IML18* were greatly induced in *C. militaris* challenged larvae at 72 and 12 h post‐infection (hpi), respectively (Fig. 6B). After fungus *O. sinensis* or *C. militaris* infection, seven genes including *IML3*, *4*, *16*, *20*, *CTL‐S5*, *S6* and *CTL‐X6* were commonly up‐regulated, and *IML7*, *9* and *CTL‐S2* were commonly down‐regulated (Fig. 6A and 6B). Expression levels of the gene encoding CTL‐X2 generally showed a two‐fold up‐regulation after infection (Fig. 6A and 6B). There are nine genes including genes encoding *IML1*, *2*, *5*, *10–13*, *15* and *22* that were all quickly activated in all conditions. No unique induction was seen in the Ringer's treated groups.

## Discussion

In this study, 32 CTLDP genes were annotated from the *T. xiaojinensis* transcriptome. The orthologues of all *T. xiaojinensis* CTL‐Ss were found in other insects (Fig. 2A) indicating that the emergence of these six genes occurred earlier than species differentiation. *T. xiaojinensis* also lacked some genes, such as the orthologues to *B. mori* CTL‐S8–S12. The *M. sexta* genome encodes 19 IMLs, *B. mori* genome encodes six IMLs and the *H. armigera* genome encodes 11 IMLs (Rao *et al*., 2015a, b; Xiong *et al*., 2015). The number of IMLs (23) in *T. xiaojinensis* is relatively high and the phylogenetic tree shows a possible lineage‐specific expansion in Figure 2B. IMLs have probably experienced evolutionary selection pressure in the ghost moth. There are only three identified CTLDPs belonging to CTL‐X subfamily (Fig. 1 and Table S3). The neighbor‐joining tree in Figure 2C revealed that these three *T. xiaojinensis* CTL‐Xs individually formed orthologues to *M. sexta* CTL‐X2, X4 and X6. The absence of *T. xiaojinensis* orthologues to other *M. sexta* CTL‐X may be attributed to length limitations of the assembled transcripts. In addition, the distinct manner of evolution between the IML family (species‐specific expansion) and CTL‐S along with the CTL‐X family (monophyletic group) indirectly reflects their potential functions in the ghost moth. It seems that members in the IML family have undergone significant evolutionary pressure leading to duplication and divergence, indicating that they are probably targets for diverse foreigners. In contrast, conservation was observed in *T. xiaojinensis* CTL‐Ss and CTL‐Xs, the orthologues of which could be found in other species, indicating that they might deal with endogenous glycans or act in cell‐cell adhesion and tissue integration.

CTL‐S had sequences that were relatively simple with only one CRD and no other detectable domains (Table S3 and Fig. 1), and appears to be more primitive than the other two subfamilies. This was illustrated in the phylogenetic tree established by 53 CTLDs from *T. xiaojinensis* (Fig. 3). There were two reasons for building the tree in Figure 2A. One reason was to reveal the evolutionary relationships among the CTL‐Ss from various species. The close relation among CTL‐S1, S2 and S3 is consistent with the information in Figure 3. The second reason was to predict potential functions of *T. xiaojinensis* CTL‐S through their relationships with other characterized CTL‐S (Table S1). Two well‐known CTLDs (AgCTL4 and MA2) in *A. gambiae* have been reported to protect *Plasmodium* parasites from melanization (Osta *et al*., 2004). *A. aegypti* mosGCTL‐1 and ‐3 are used as receptors or attachment factors to facilitate West Nile virus (WNV) and dengue virus‐2 (DENV‐2) invasion, respectively (Cheng *et al*., 2010; Liu *et al*., 2014). Five CTL‐Ss from *Armigeres subalbatus* were tested for their roles in innate immunity and AsCTLGA5 was involved in resistance against *Escherichia coli* by rna interference (RNAi) experiments (Shi *et al*., 2014). However, none of these well‐studied CTL‐S exhibited close connections with *T. xiaojinensis* CTL‐S. They all clustered together reflecting mosquito‐specific expansion (Fig. 2A). These receptors have been exposed to significant evolutionary pressure and the distinctive extension in some members might be driven by particular microbes. In Lepidoptera, the characteristics of only one CTL‐S have been determined, namely *B. mori* CTL‐S4. This had no ability to bind microorganisms and its function is still unknown (Takase *et al*., 2009). Therefore, determining the roles of Lepidopteran CTL‐S will require more molecular and biochemical experiments.

Unlike mammalian or Dipteran members of the CTL‐S family, Lepidoptera have enlarged their own IML family during evolution. An apparent lineage‐specific expansion of IML is seen in many Lepidoptera. Based on the characteristics of IMLs from *M. sexta*, *B. mori*, *Antheraea pernyi* and *H. armigera*, these proteins are mainly involved in defense responses, such as binding to intruders and inducing phagocytosis, agglutination, encapsulation, PPO activation and melanization (Table S2). The evolved pattern of IMLs and known functions of some receptors suggest that IMLs may be primary targets for pathogens and parasites. Therefore, they may have evolved into tandem‐CRD forms to enhance binding affinity and extend their spectrum of recognition. *B. mori* IML6, *M. sexta* IML1, *H. armigera* CTL3, *Spodoptera exigua* LL3 and *Papilio xuthus* XP_011552083.1 (Genbank accession number) formed an orthologous group and so did *B. mori* IML4, *M. sexta* IML19, *Danaus plexippus* CTL18 and *P. xuthus* XP_013176126.1 (Fig. 2B). However, no orthologues to those IMLs were identified in *T. xiaojinensis* (Fig. 2B) indicating that these genes emerged after divergence of *B. mori*, *M. sexta*, *H. armigera* and butterflies from primitive insect groups. In the end of IML‐A in *H. armigera* there is a conserved PXXC motif, while in the end of IML‐B there is a conserved FXCE motif (Wang *et al*., 2012). Most dual CRDs in *T. xiaojinensis* IMLs are consistent with that rule, except IML2B, 5B, 10B, 19B and IML17A (Fig. 4). We found another interesting property in their compositions. There is a conserved glycine (Gly) behind the Cys‐4 of IML‐B and the residues at the first site after Cys‐4 of IML‐A are Val/Ile/Leu/Ala. This feature is not only seen in *T. xiaojinensis* IMLs but also in other identified IMLs (Rao *et al*., 2015a, b; Xiong *et al*., 2015). It is known that the four to six Cys residues in CTLDs are responsible for formation of disulfide bonds, and thus motif signatures around Cys, conserved in most IMLs, may have important effects on protein sorting. Uncovering the characteristics of IML sequences will help distinguish IML from CTL‐S and CTL‐X. Under some conditions there is an incomplete amino acid sequence with only one CTLD, but it probably belongs to the IML subfamily, like *T. xiaojinensis* IML18 and IML21 (Fig. 4).

CTL‐X represents a subfamily of CTLDPs with CTLDs and other identically conserved domains. In *T. xiaojinensis*, there were three transcripts encoding proteins belonging to this subfamily (Table S3 and Fig. 1). Although the N‐ or C‐terminus is absent in *T. xiaojinensis* CTL‐X2, 4 and 6, the matched parts of these CTLDPs showed high identity (77.85%, 58.25% and 70.87%, respectively) to CTL‐X2, 4 and 6 from *M. sexta*. The phylogenetic tree in Figure 2C revealed monophyletic groups among CTL‐X orthologues from other insects, indicating these receptors might have similar functions in different species. The insect CTL‐Xs are mainly associated with cell adhesion and developmental regulation. It is reported that the *D. melanogaster furrowed* gene in CTL‐X2 group encodes a protein, similar to vertebrate selectins, and mutation of complement control protein regions in the protein interrupted development of sensory organs (Leshko‐Lindsay & Corces, 1997). Genetic, molecular and biochemical features of *D. melanogaster* contactin (an orthologue to *Thitarodes* CTL‐X4) were studied by Faivre‐Sarrailh *et al*. (2004) who demonstrated that this cell adhesion molecule is required for organization of septate junctions involved in formation and maintenance of charge and size selective barriers. Although the functions of many CTL‐X group members have been reported in *D. melanogaster*, the roles of CTLDs are unknown. Characteristics of most identified CTL‐X from Lepidoptera have not been investigated, and their functions such as properties of their CTLDs are unknown.

In addition to phylogenetic analysis, binding ligands and structural models for *T. xiaojinensis* CTLDPs were also analyzed. Except for one CRD in CTL‐S4, the remaining CRDs in *T. xiaojinensis* were all identified as having at least one Ca^2+^‐association site (Table S5). This proportion is quite different from *M. sexta* and *B. mori*. A total of 56 CTLDs were investigated in *M. sexta* and only 18 models contained one or two Ca^2+^ ions (Rao *et al*., 2015a). In *B. mori*, nearly 70% of the CTLDs were expected to interact with Ca^2+^ (Rao *et al*., 2015b). This difference suggests that the sites for Ca^2+^ interaction in CTLDPs are variable in different Lepidoptera species. It is worth noting that a type II antifreeze protein from the liver of smelt (*Osmerus mordax*) is homologous to CTLDP and its antifreeze activity was responsive to Ca^2+^ (Ewart *et al*., 1992). *T. xiaojinensis* is adapted to the cold alpine meadows of Xiaojin County, where the mean annual temperature is –3.2°C (Zhu *et al*., 2016). *B. mori* and *M. sexta*, as model insects, are reared in the laboratory at 25°C. Therefore, the preservation of Ca^2+^‐binding sites for most CTLDPs may be an environmental adaptation of *T. xiaojinensis*. In asialoglycoprotein and macrophage mannose receptors, missing Ca^2+^ causes transformation of the CRD region and this results in release of the bound ligands (Loeb & Drickamer, 1988). There is evidence that Ca^2+^ also plays a negative role in association with ligands. Plasminogen cannot interact with tetranectin (a CTLDP in humans) until Ca^2+^ removes it from the binding site (Graversen *et al*., 1998). We believe that *T. xiaojinensis* uses Ca^2+^ ions as a mediator to flexibly control the recognition of CTLDPs to other ligands.

Expression profiles of CTLDPs in the fat body (a major immune tissue for insects) of *T. xiaojinensis* after different stimulations provide some indications about their functions. *T. xiaojinensis* CTL‐Xs are not expected to participate in immune defense, since their FPKM values are all very low (Table S6). Considering the characteristics of *D. melanogaster* CTL‐X, *T. xiaojinensis* CTL‐Xs may be involved in cell‐cell adhesion or tissue integration. *T. xiaojinensis* CTL‐S1–S6 are also expressed at low levels in all untreated and treated larvae (Table S6), indicating that their targets are probably endogenous sugars or sugar derivatives rather than microbes. To *T. xiaojinensis*, pathogenicity of *O. sinensis* is different from pathogenicity of *C. militaris* and *E. cloacae*. After infection with *C. militaris* or *E. cloacae*, the larvae die within several days. However, *O. sinensis* can infect larvae for a long time, ranging from several months to 1 year (Meng *et al*., 2015). As previously noted, IMLs probably play significant roles in binding a variety of microbes and activating immune responses in *T. xiaojinensis*. Therefore, the inducible ability of *T. xiaojinensis* IMLs could provide clues to decipher distinct reactions that are elicited by different challenges. IML8 is likely an important target for *C. militaris*, as its mRNA level was greatly up‐regulated in larvae infected by the fungus for 72 h (Fig. 6B). IML14 is likely a specific receptor for *O. sinensis*, as its significant up‐regulation was only detected in larvae infected by *O. sinensis* for 72 h (Fig. 6B). It would be useful to compare functional features of IML14 and 8 in *T. xiaojinensis* immunity.

In conclusion, 32 CTLDP genes identified from *T. xiaojinensis* were divided into three subfamilies and renamed. Phylogenetic analysis revealed evolutionary diversification of these genes. Gene duplication and merging may have resulted in IMLs possessing two CTLDs and natural selection‐based expansion of this subfamily in *T. xiaojinensis*. In addition, most of the identified CTLDPs in *T. xiaojinensis* had Ca^2+^ affinity possibly to regulate interactions of these receptors with other ligands and to protect them from freezing. Structural comparisons revealed that the structure of the second loop could also affect the specificity of binding ligands. CTLDP gene expression profiles in response to different immune challenges provide useful information for future functional studies in species of primitive Lepidoptera.

## Disclosure

The authors declare no conflicts of interests.

## Supporting information


**Fig. S1**. Structure‐based sequence alignment of *Thitarodes xiaojinensis* C‐type lectin domains (CTLDs) and the CTLD in human DC‐SIGNR. Multiple sequence alignment was carried out by MUSCLE, a module in MEGA 6.0, and depicted by ESPript. Identical residues are marked in red and similar residues are in yellow. The carbohydrate‐recognition motifs are indicated by the box enclosed with a red line.

**Table S1**. Functions of the C‐type lectin domain proteins (CTLDPs) with one carbohydrate‐recognition domain (CRD) in Dipteran.
**Table S2**. Functions of the C‐type lectin domain proteins (CTLDPs) with two carbohydrate‐recognition domains (CRDs) in Lepidoptera.
**Table S3**. Features of 32 *Thitarodes xiaojinensis* C‐type lectin domain proteins (CTLDPs).
**Table S4**. Primers for 6 genes encoding C‐type lectin domain proteins (CTLDPs).
**Table S5**. Structural characteristics of 53 C‐type lectin domains (CTLDs) in *Thitarodes xiaojinensis*.
**Table S6**. FPKM values of 32 *Thitarodes xiaojinensis* genes encoding C‐type lectin domain proteins (CTLDPs) in ten libraries.

## References

[ins12564-bib-0001] Ao, J. , Ling, E. and Yu, X.Q. (2007) *Drosophila* C‐type lectins enhance cellular encapsulation. Molecular Immunology, 44, 2541–2548. 17287021 10.1016/j.molimm.2006.12.024 PMC1876673

[ins12564-bib-0002] Cheng, G. , Cox, J. , Wang, P. , Krishnan, M.N. , Dai, J. , Qian, F. *et al* (2010) A C‐type lectin collaborates with a CD45 phosphatase homolog to facilitate west nile virus infection of mosquitoes. Cell, 142, 714–725. 20797779 10.1016/j.cell.2010.07.038 PMC2954371

[ins12564-bib-0003] Ao, J. , Ling, E. and Yu, X.Q. (2007) *Drosophila* C‐type lectins enhance cellular encapsulation. Molecular Immunology, 44, 2541–2548. 17287021 10.1016/j.molimm.2006.12.024 PMC1876673

[ins12564-bib-0004] Cheng, G. , Cox, J. , Wang, P. , Krishnan, M.N. , Dai, J. , Qian, F. *et al* (2010) A C‐type lectin collaborates with a CD45 phosphatase homolog to facilitate west nile virus infection of mosquitoes. Cell, 142, 714–725. 20797779 10.1016/j.cell.2010.07.038 PMC2954371

[ins12564-bib-0005] Evans, J.D. , Aronstein, K. , Chen, Y.P. , Hetru, C. , Imler, J.L. , Jiang, H. *et al* (2006) Immune pathways and defence mechanisms in honey bees *Apis mellifera* . Insect Molecular Biology, 15, 645–656. 17069638 10.1111/j.1365-2583.2006.00682.x PMC1847501

[ins12564-bib-0006] Ewart, K.V. , Rubinsky, B. and Fletcher, G.L. (1992) Structural and functional similarity between fish antifreeze proteins and calcium‐dependent lectins. Biochemical and Biophysical Research Communications, 185, 335–340. 1599470 10.1016/s0006-291x(05)90005-3

[ins12564-bib-0007] Faivre‐Sarrailh, C. , Banerjee, S. , Li, J. , Hortsch, M. , Laval, M. and Bhat, M.A. (2004) *Drosophila* contactin, a homolog of vertebrate contactin, is required for septate junction organization and paracellular barrier function. Development, 131, 4931–4942. 15459097 10.1242/dev.01372

[ins12564-bib-0008] Graversen, J.H. , Lorentsen, R.H. , Jacobsen, C. , Moestrup, S.K. , Sigurskjold, B.W. , Thogersen, H.C. *et al* (1998) The plasminogen binding site of the C‐type lectin tetranectin is located in the carbohydrate recognition domain, and binding is sensitive to both calcium and lysine. Journal of Biological Chemistry, 273, 29241–29246. 9786936 10.1074/jbc.273.44.29241

[ins12564-bib-0009] Koizumi, N. , Imai, Y. , Morozumi, A. , Imamura, M. , Kadotani, T. , Yaoi, K. *et al* (1999) Lipopolysaccharide‐binding protein of *Bombyx mori* participates in a hemocyte‐mediated defense reaction against gram‐negative bacteria. Journal of Insect Physiology, 45, 853–859. 12770298 10.1016/s0022-1910(99)00069-4

[ins12564-bib-0010] Lemaitre, B. and Hoffmann, J.A. (2007) The host defense of *Drosophila melanogaster* . Annual Review of Immunology, 25, 697–743. 10.1146/annurev.immunol.25.022106.141615 17201680

[ins12564-bib-0011] Leshko‐Lindsay, L.A. and Corces, V.G. (1997) The role of selectins in *Drosophila* eye and bristle development. Development, 124, 169–180. 9006078 10.1242/dev.124.1.169

[ins12564-bib-0012] Liu, Y. , Zhang, F.C. , Liu, J.Y. , Xiao, X.P. , Zhang, S.Y. , Qin, C.F. *et al* (2014) Transmission‐blocking antibodies against mosquito C‐type lectins for dengue prevention. PLoS Pathogens, 10, e1003931. 24550728 10.1371/journal.ppat.1003931 PMC3923773

[ins12564-bib-0013] Loeb, J.A. and Drickamer, K. (1988) Conformational changes in the chicken receptor for endocytosis of glycoproteins. Modulation of ligand‐binding activity by Ca^2+^ and pH. Journal of Biological Chemistry, 263, 9752–9760. 3290213

[ins12564-bib-0014] Meng, Q. , Yu, H.Y. , Zhang, H. , Zhu, W. , Wang, M.L. , Zhang, J.H. *et al* (2015) Transcriptomic insight into the immune defenses in the ghost moth, *Hepialus xiaojinensis*, during an *Ophiocordyceps sinensis* fungal infection. Insect Biochemistry and Molecular Biology, 64, 1–15. 26165779 10.1016/j.ibmb.2015.06.014

[ins12564-bib-0015] Nielsen, E.S. , Robinson, G.S. and Wagner, D.L. (2000) Ghost‐moths of the world: a global inventory and bibliography of the Exoporia (Mnesarchaeoidea and Hepialoidea) (Lepidoptera). Journal of Natural History, 34, 823–878.

[ins12564-bib-0016] Osta, M.A. , Christophides, G.K. and Kafatos, F.C. (2004) Effects of mosquito genes on *Plasmodium* development. Science, 303, 2030–2032. 15044804 10.1126/science.1091789

[ins12564-bib-0017] Pal, S. and Wu, L.P. (2009) Lessons from the fly: pattern recognition in *Drosophila melanogaster* . Advances in Experimental Medicine and Biology, 653, 162–174. 19799118 10.1007/978-1-4419-0901-5_11

[ins12564-bib-0018] Probert, F. , Whittaker, S.B. , Crispin, M. , Mitchell, D.A. and Dixon, A.M. (2013) Solution NMR analyses of the C‐type carbohydrate recognition domain of DC‐SIGNR protein reveal different binding modes for HIV‐derived oligosaccharides and smaller glycan fragments. Journal of Biological Chemistry, 288, 22745–22757. 23788638 10.1074/jbc.M113.458299 PMC3829359

[ins12564-bib-0019] Rao, X.J. , Cao, X. , He, Y. , Hu, Y. , Zhang, X. , Chen, Y.R. *et al* (2015a) Structural features, evolutionary relationships, and transcriptional regulation of C‐type lectin‐domain proteins in *Manduca sexta* . Insect Biochemistry and Molecular Biology, 62, 75–85. 25554596 10.1016/j.ibmb.2014.12.006 PMC4476918

[ins12564-bib-0020] Rao, X.J. , Shahzad, T. , Liu, S. , Wu, P. , He, Y.T. , Sun, W.J. *et al* (2015b) Identification of C‐type lectin‐domain proteins (CTLDPs) in silkworm *Bombyx mori* . Developmental & Comparative Immunology, 53, 328–338. 26187302 10.1016/j.dci.2015.07.005

[ins12564-bib-0021] Ren, J. , Wen, L.P. , Gao, X.J. , Jin, C.J. , Xue, Y. and Yao, X.B. (2009) DOG 1.0: illustrator of protein domain structures. Cell Research, 19, 271–273. 19153597 10.1038/cr.2009.6

[ins12564-bib-0022] Roy, A. , Kucukural, A. and Zhang, Y. (2010) I‐TASSER: a unified platform for automated protein structure and function prediction. Nature Protocols, 5, 725–738. 20360767 10.1038/nprot.2010.5 PMC2849174

[ins12564-bib-0023] Shi, X.Z. , Kang, C.J. , Wang, S.J. , Zhong, X. , Beerntsen, B.T. and Yu, X.Q. (2014) Functions of *Armigeres subalbatus* C‐type lectins in innate immunity. Insect Biochemistry and Molecular Biology, 52, 102–114. 25014898 10.1016/j.ibmb.2014.06.010 PMC4143534

[ins12564-bib-0024] Shin, S.W. , Zou, Z. and Raikhel, A.S. (2011) A new factor in the *Aedes aegypti* immune response: CLSP2 modulates melanization. EMBO Reports, 12, 938–943. 21760616 10.1038/embor.2011.130 PMC3166456

[ins12564-bib-0025] Takase, H. , Watanabe, A. , Yoshizawa, Y. , Kitami, M. and Sato, R. (2009) Identification and comparative analysis of three novel C‐type lectins from the silkworm with functional implications in pathogen recognition. Developmental & Comparative Immunology, 33, 789–800. 19201380 10.1016/j.dci.2009.01.005

[ins12564-bib-0026] Tamura, K. , Stecher, G. , Peterson, D. , Filipski, A. and Kumar, S. (2013) MEGA6: Molecular Evolutionary Genetics Analysis version 6.0. Molecular Biology and Evolution, 30, 2725–2729. 24132122 10.1093/molbev/mst197 PMC3840312

[ins12564-bib-0027] Tanji, T. , Ohashi‐Kobayashi, A. and Natori, S. (2006) Participation of a galactose‐specific C‐type lectin in *Drosophila* immunity. Biochemical Journal, 396, 127–138. 16475980 10.1042/BJ20051921 PMC1450005

[ins12564-bib-0028] Thompson, J.D. , Gibson, T.J. , Plewniak, F. , Jeanmougin, F. and Higgins, D.G. (1997) The CLUSTAL_X windows interface: flexible strategies for multiple sequence alignment aided by quality analysis tools. Nucleic Acids Research, 25, 4876–4882. 9396791 10.1093/nar/25.24.4876 PMC147148

[ins12564-bib-0029] van Vliet, S.J. , Saeland, E. and van Kooyk, Y. (2008) Sweet preferences of MGL: carbohydrate specificity and function. Trends in Immunology, 29, 83–90. 18249034 10.1016/j.it.2007.10.010

[ins12564-bib-0030] Wang, J.L. , Liu, X.S. , Zhang, Q. , Zhao, H.B. and Wang, Y.F. (2012) Expression profiles of six novel C‐type lectins in response to bacterial and 20E injection in the cotton bollworm (*Helicoverpa armigera*). Developmental & Comparative Immunology, 37, 221–232. 22516747 10.1016/j.dci.2012.04.004

[ins12564-bib-0031] Wang, R.J. , Lin, Z. , Jiang, H. , Li, J.C. , Saha, T.T. , Lu, Z.Y. *et al* (2017) Comparative analysis of peptidoglycan recognition proteins in endoparasitoid wasp *Microplitis mediator* . Insect Science, 24, 2–16. 26549814 10.1111/1744-7917.12290

[ins12564-bib-0032] Wang, X.L. , Zhang, J.H. , Chen, Y. , Ma, Y.L. , Zou, W.J. , Ding, G.Y. *et al* (2013) A novel pattern recognition protein of the Chinese oak silkmoth, *Antheraea pernyi*, is involved in the pro‐PO activating system. Biochemistry and Molecular Biology Reports, 46, 358–363. 10.5483/BMBRep.2013.46.7.009 PMC4133915 23884102

[ins12564-bib-0033] Wang, Y.H. , Hu, Y. , Xing, L.S. , Jiang, H. , Hu, S.N. , Raikhel, A.S. *et al* (2015) A critical role for CLSP2 in the modulation of antifungal immune response in mosquitoes. PLoS Pathogens, 11, e1004931. 26057557 10.1371/journal.ppat.1004931 PMC4461313

[ins12564-bib-0034] Watanabe, A. , Miyazawa, S. , Kitami, M. , Tabunoki, H. , Ueda, K. and Sato, R. (2006) Characterization of a novel C‐type lectin, *Bombyx mori* multibinding protein, from the *B. mori* hemolymph: mechanism of wide‐range microorganism recognition and role in immunity. Journal of Immunology, 177, 4594–4604. 10.4049/jimmunol.177.7.4594 16982897

[ins12564-bib-0035] Waterhouse, R.M. , Kriventseva, E.V. , Meister, S. , Xi, Z. , Alvarez, K.S. , Bartholomay, L.C. *et al* (2007) Evolutionary dynamics of immune‐related genes and pathways in disease‐vector mosquitoes. Science, 316, 1738–1743. 17588928 10.1126/science.1139862 PMC2042107

[ins12564-bib-0036] Wu, C. , Charoensapsri, W. , Nakamura, S. , Tassanakajon, A. , Söderhäll, I. and Söderhäll, K. (2013) An MBL‐like protein may interfere with the activation of the proPO‐system, an important innate immune reaction in invertebrates. Immunobiology, 218, 159–168. 22459272 10.1016/j.imbio.2012.02.011

[ins12564-bib-0037] Xiong, G.H. , Xing, L.S. , Lin, Z. , Saha, T.T. , Wang, C. , Jiang, H. *et al* (2015) High throughput profiling of the cotton bollworm *Helicoverpa armigera* immunotranscriptome during the fungal and bacterial infections. BMC Genomics, 16, 321. 26001831 10.1186/s12864-015-1509-1 PMC4490664

[ins12564-bib-0038] Yang, J. , Roy, A. and Zhang, Y. (2013) Protein‐ligand binding site recognition using complementary binding‐specific substructure comparison and sequence profile alignment. Bioinformatics, 29, 2588–2595. 23975762 10.1093/bioinformatics/btt447 PMC3789548

[ins12564-bib-0039] Yu, X.Q. , Tracy, M.E. , Ling, E. , Scholz, F.R. and Trenczek, T. (2005) A novel C‐type immulectin‐3 from *Manduca sexta* is translocated from hemolymph into the cytoplasm of hemocytes. Insect Biochemistry and Molecular Biology, 35, 285–295. 15763465 10.1016/j.ibmb.2005.01.004

[ins12564-bib-0040] Yu, X.Q. , Gan, H. and Kanost, M.R. (1999) Immulectin, an inducible C‐type lectin from an insect, *Manduca sexta*, stimulates activation of plasma prophenol oxidase. Insect Biochemistry and Molecular Biology, 29, 585–597. 10436935 10.1016/s0965-1748(99)00036-3

[ins12564-bib-0041] Yu, X.Q. , Ling, E. , Tracy, M.E. and Zhu, Y. (2006) Immulectin‐4 from the tobacco hornworm *Manduca sexta* binds to lipopolysaccharide and lipoteichoic acid. Insect Molecular Biology, 15, 119–128. 16640722 10.1111/j.1365-2583.2006.00618.x

[ins12564-bib-0042] Zelensky, A.N. and Gready, J.E. (2005) The C‐type lectin‐like domain superfamily. FEBS Journal, 272, 6179–6217. 16336259 10.1111/j.1742-4658.2005.05031.x

[ins12564-bib-0043] Zhang, D.L. and Tu, Y.Q. (2015) The determination of *Thitarodes xiaojinensis* ecotype. Journal of Environmental Entomology, 37, 1055–1059.

[ins12564-bib-0044] Zhu, W. , Zhang, H. , Li, X. , Meng, Q. , Shu, R.H. , Wang, M.L. *et al* (2016) Cold adaptation mechanisms in the ghost moth *Hepialus xiaojinensis*: Metabolic regulation and thermal compensation. Journal of Insect Physiology, 85, 76–85. 26585102 10.1016/j.jinsphys.2015.11.008

[ins12564-bib-0045] Zou, Z. , Evans, J.D. , Lu, Z. , Zhao, P. , Williams, M. , Sumathipala, N. *et al* (2007) Comparative genomic analysis of the *Tribolium* immune system. Genome Biology, 8, R177. 17727709 10.1186/gb-2007-8-8-r177 PMC2375007

