# Supplementary material for: Comparative analysis of C‐type lectin domain proteins in the ghost moth, Thitarodes xiaojinensis (Lepidoptera: Hepialidae)
Source: Insect Sci. 2018 Feb 22;26(3):453–65. doi: 10.1111/1744-7917.12564 (PMC7379682; doi:10.1111/1744-7917.12564)
Supplement: Supplementary file 1 — Fig. S1. Structure‐based sequence alignment of Thitarodes xiaojinensis C‐type lectin domains (CTLDs) and the CTLD in human DC‐SIGNR. Multiple sequence alignment was carried out by MUSCLE, a module in MEGA 6.0, and depicted by ESPript. Identical residues are marked in red and similar residues are in yellow. The carbohydrate‐recognition motifs are indicated by the box enclosed with a red line. [file INS-26-453-s001.docx]

Supplementary Figure


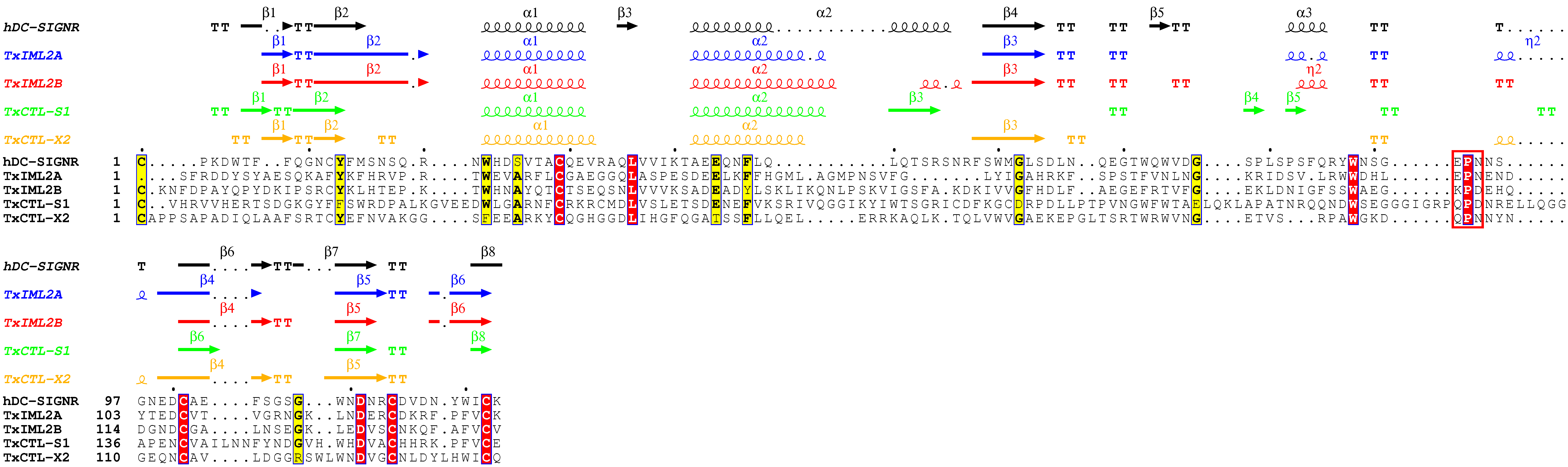


Fig. S1. Structure-based sequence alignment of *T. xiaojinensis* CTLDs and the CTLD in human DC-SIGNR. Multiple sequence alignment was carried out by MUSCLE, a module in MEGA 6.0, and depicted by ESPript. Identical residues are marked in red and similar redidues are in yellow. The carbohydrate-recognition motifs are indicated by the box enclosed with red line.
