# Supplementary material for: Comparative analysis of C‐type lectin domain proteins in the ghost moth, Thitarodes xiaojinensis (Lepidoptera: Hepialidae)
Source: Insect Sci. 2018 Feb 22;26(3):453–65. doi: 10.1111/1744-7917.12564 (PMC7379682; doi:10.1111/1744-7917.12564)
Supplement: Supplementary file 2 — Table S1. Functions of the C‐type lectin domain proteins (CTLDPs) with one carbohydrate‐recognition domain (CRD) in Dipteran. Table S2. Functions of the C‐type lectin domain proteins (CTLDPs) with two carbohydrate‐recognition domains (CRDs) in Lepidoptera. Table S3. Features of 32 Thitarodes xiaojinensis C‐type lectin domain proteins (CTLDPs). Table S4. Primers for 6 genes encoding C‐type lectin domain proteins (CTLDPs). Table S5. Structural characteristics of 53 C‐type lectin domains (CTLDs) in Thitarodes xiaojinensis. Table S6. FPKM values of 32 Thitarodes xiaojinensis genes encoding C‐type lectin domain proteins (CTLDPs) in ten libraries. [file INS-26-453-s002.pdf]

Table S1. Functions of the CTLDPs with one CRD in Dipteran

Name[a]	Tissue[b]					Agglutination[c]	Inducibility[c]			binding[d]	proPO activation	Melanization	Reference
	Ep	FB	Hc	Mg	others		Bacteria	fungi	others				
DL1-3, Dm lectin-24A	lectin-24A	1-3, lectin-24A	1, 3, lectin-24A	lectin-24A	SG, tracheae (1);	Ec (1-3);	-	-	parasitic wasps (lectin-24A)	galactose (1-3); - (lectin-24A)	-	Yes (2,3); - (1, lectin-24A)	(Ao et al., 2007; Keebaugh and Schlenke, 2012; Tanji et al., 2006)
AgCTL4, MA2	-	-	-	-	-	-	Ec, Sa	-	-	-	-	Inhibitor	(Osta et al., 2004; Schnitger et al., 2009)
AamosGCTL-1, 3, CLSP1, 2	-	CLSP1, 2	No (CLSP1, 2)	mosGCTL-1	SG (mosGCTL-1,)	Bb (CLSP2)	Ecl (CLSP1, 2)	Bb (CLSP1, 2)	WNV (mosGCTL-1); DENV-2 (mosGCTL-3); Pg (CLSP2)	WNV envelope protein (mosGCTL-1); DENV-2 envelope protein (mosGCTL-3)	Inhibitor (CLSP2)	-	(Cheng et al., 2010; Liu et al., 2014; Shin et al., 2011; Wang et al., 2015)
AsCTLMA11, 15, GA5, CTL15, 16	MA11, 15, GA5, CTL15, 16	MA11, 15, GA5, CTL15, 16	MA11, 15, GA5, CTL15, 16	MA11, GA5, CTL16	MT (MA11, 15, GA5, CTL15, 16)	Sa (MA15, GA5, CTL15), Ml (MA15); Bc (GA5, CTL15); Bs, Ec, Sm, Pa (CTL15); No (CTL16);	Ec (MA11, 15, GA5, CTL15); Ml (GA5, CTL15, 16)	-	-	LPS, PG, LTA, mannan,	-	-	(Shi et al., 2014)

[a] DL, Drosophila lectin; Dm, *Drosophila melanogaster*; Ag, *Anopheles gambiae*; Aa, *Aedes aegypti*; As, *Armigeres subalbatus*;

[b] Ep, epidermis; FB, fat body; Hc, hemocyte; Mg, midgut; SG, silk gland; MT, Malpighian tubule;

[c] Ml, *Micrococcus luteus*; Sa, *Staphylococcus aureus*; Bs, *Bacillus subtilis*; Bc, *Bacillus cereus*; Ec, *Escherichia coli*; Sm, *Serratia marcescens*; Pa, *Pseudomonas*

aeruginosa; Sc, *Saccharomyces cerevisiae*; WNV, West Nile virus; Ecl, *Enterobacter cloacae*; Bb, *Beauveria bassiana*; Pg, *Plasmodium gallinaceum*;
[d] LPS, lipopolysaccharide; PG, peptidoglycan; LTA, lipoteichoic acid;

Ao, J., Ling, E., Yu, X.Q., 2007. *Drosophila* C-type lectins enhance cellular encapsulation. Mol. Immunol. 44, 2541-2548.

Cheng, G., Cox, J., Wang, P., Krishnan, M.N., Dai, J., Qian, F., Anderson, J.F., Fikrig, E., 2010. A C-Type Lectin Collaborates with a CD45 Phosphatase Homolog to Facilitate West Nile Virus Infection of Mosquitoes. Cell 142, 714-725.

Keebaugh, E.S., Schlenke, T.A., 2012. Adaptive evolution of a novel *Drosophila* lectin induced by parasitic wasp attack. Mol. Biol. Evol. 29, 565-577.

Liu, Y., Zhang, F., Liu, J., Xiao, X., Zhang, S., Qin, C., Xiang, Y., Wang, P., Cheng, G., 2014. Transmission-blocking antibodies against mosquito C-type lectins for dengue prevention. PLoS Pathog 10, e1003931.

Osta, M.A., Christophides, G.K., Kafatos, F.C., 2004. Effects of mosquito genes on *Plasmodium* development. Science 303, 2030-2032.

Schnitger, A.K., Yassine, H., Kafatos, F.C., Osta, M.A., 2009. Two C-type lectins cooperate to defend *Anopheles gambiae* against Gram-negative bacteria. J. Biol. Chem. 284, 17616-17624.

Shi, X.-Z., Kang, C.-J., Wang, S.-J., Zhong, X., Beerntsen, B.T., Yu, X.-Q., 2014. Functions of *Armigeres subalbatus* C-type lectins in innate immunity. Insect Biochem. Mol. Biol. 52, 102-114.

Shin, S.W., Zou, Z., Raikhel, A.S., 2011. A new factor in the *Aedes aegypti* immune response: CLSP2 modulates melanization. EMBO Rep 12, 938-943.

Tanji, T., Ohashi-Kobayashi, A., Natori, S., 2006. Participation of a galactose-specific C-type lectin in *Drosophila* immunity. Biochem. J. 396, 127-138.

Wang, Y.H., Hu, Y., Xing, L.S., Jiang, H., Hu, S.N., Raikhel, A.S., Zou, Z., 2015. A Critical Role for CLSP2 in the Modulation of Antifungal Immune Response in Mosquitoes. PLoS Pathog 11, e1004931.

Table S2. Functions of the CTLDPs with two CRDs in Lepidoptera

Name[a]	Tissue[b]					Agglutination[c]	Inducibility[c]			binding[d]	proPO activation	Melanization	Reference
	Ep	FB	Hc	Mg	others		Bacteria	fungi	others				
MsIML1-4	-	1-4	3, 4	1-4	MT (1-4); testis, ovary (1-3);	Ec, Sa, Sc, (1, 4);	Ec, Ml (1-4);	Sc (1-4);	-	Mannan (2), laminarin (2-4), LPS (1-4), LTA (2-4), lipid A (2), PG (2); - (1)	Yes (1, 2, 4); -(3)	Yes (2, 4); No (1, 3)	(Ling and Yu, 2006; Rao et al., 2015a; Yu et al., 2005; Yu et al., 1999; Yu et al., 2006)
BmIML1, 3-6	-	1, 3, 5, 6	1, 3, 5, 6	1	MT (1, 5, 6); SG (1, 5); Ig, (1,6); testis, ovary (1,4-6);	Ml, Sl, Ca (1); Ec (rough) (1, 3); Smi (rough) (3);	Ec (smooth) (4); Ml (4, 5);	-	-	Ec (rough), Sm (1, 4, 5); Ec (smooth) (4); Pv (3), Ml (1,3); Sc, Ca, Sl (1); LPS, lipid A (3); - (6);	-	Yes (3)	(Koizumi et al., 1999; Koizumi et al., 1997; Rao et al., 2015b; Takase et al., 2009; Watanabe et al., 2006)
HaCTL1-8	3, 6, 7, 8	1, 3-8	1, 2, 6, 7	1, 3-8	-	Ec, Sa (2,3, 7); Bt, Pa, Kn, Pp, Ca (2); - (1, 4-6, 8)	Ec (1-8); Sa (1, 4-8);	Pp (1)	HaNPV (1), 20E (1, 3-6, 8);	PG (1-3, 7), Maltose (3); trehalose , LPS (3, 7); mannose (1, 2, 7); galactose, sucrose (1, 2); curdlan (1); - (4-6, 8);	-	Yes(7); - (1-6, 8);	(Chai et al., 2008; Tian et al., 2009; Wang et al., 2012; Wang et al., 2014)
ApCTL	No	Yes	No	Yes	-	Ec, Sa, Sc	-	-	a mixture of killed Ec, Sa,	LPS, laminarin, mannan, PG, LTA	Yes	-	(Xialu et al., 2013)

Ca and Sc

[a] Ms, *Manduca sexta*; Bm, *Bombyx mori*; Ha, *Helicoverpa armigera*; Ap, *Antheraea pernyi*;

[b] Ep, epidermis; FB, fat body; Mg, midgut; Hc, hemocyte; SG, silk gland; MT, Malpighian tubule; Ig, integument;

[c] Ec, *Escherichia coli*; Ml, *Micrococcus luteus*; Sa, *Staphylococcus aureus*; Ca, *Candida albicans*; Sc, *Saccharomyces cerevisiae*; Sm, *Serratia marcescens*; Smi, Salmonella Minnesota; Sl, *Saccharomyces ludwigii*; Pv, *Proteus vulgaris*; Va, *Vibrio anguillarum*; Bt, *Bacillus thuringiensis*; Pp, *Pichia pastoris*; Pa, *Pseudomonas aeruginosa*; Kn, *Klebsiella pneumoniae*; HaNPV, *H. armigera* nuclear polyhedrosis virus; 20E, 20-hydroxyecdysone;

[d] LPS, lipopolysaccharide; PG, peptidoglycan; LTA, lipoteichoic acid;

Chai, L.Q., Tian, Y.Y., Yang, D.T., Wang, J.X., Zhao, X.F., 2008. Molecular cloning and characterization of a C-type lectin from the cotton bollworm, *Helicoverpa armigera*. Dev. Comp. Immunol. 32, 71-83.

Koizumi, N., Imai, Y., Morozumi, A., Imamura, M., Kadotani, T., Yaoi, K., Iwahana, H., Sato, R., 1999. Lipopolysaccharide-binding protein of *Bombyx mori* participates in a hemocyte-mediated defense reaction against gram-negative bacteria. J. Insect Physiol. 45, 853-859.

Koizumi, N., Morozumi, A., Imamura, M., Tanaka, E., Iwahana, H., Sato, R., 1997. Lipopolysaccharide-binding proteins and their involvement in the bacterial clearance from the hemolymph of the silkworm *Bombyx mori*. Eur. J. Biochem. 248, 217-224.

Ling, E., Yu, X.-Q., 2006. Cellular encapsulation and melanization are enhanced by immulectins, pattern recognition receptors from the tobacco hornworm *Manduca sexta*. Dev. Comp. Immunol. 30, 289-299.

Rao, X.-J., Cao, X., He, Y., Hu, Y., Zhang, X., Chen, Y.-R., Blissard, G., Kanost, M.R., Yu, X.-Q., Jiang, H., 2015a. Structural features, evolutionary relationships, and transcriptional regulation of C-type lectin-domain proteins in *Manduca sexta*. Insect Biochem. Mol. Biol. 62, 75-85.

Rao, X.-J., Shahzad, T., Liu, S., Wu, P., He, Y.-T., Sun, W.-J., Fan, X.-Y., Yang, Y.-F., Shi, Q., Yu, X.-Q., 2015b. Identification of C-type lectin-domain proteins (CTLDPs) in silkworm *Bombyx mori*. Dev. Comp. Immunol. 53, 328-338.

Takase, H., Watanabe, A., Yoshizawa, Y., Kitami, M., Sato, R., 2009. Identification and comparative analysis of three novel C-type lectins from the silkworm with functional implications in pathogen recognition. Dev. Comp. Immunol. 33, 789-800.

Tian, Y.Y., Liu, Y., Zhao, X.F., Wang, J.X., 2009. Characterization of a C-type lectin from the cotton bollworm, *Helicoverpa armigera*. Dev. Comp. Immunol. 33, 772-779.

Wang, J.L., Liu, X.S., Zhang, Q., Zhao, H.B., Wang, Y.F., 2012. Expression profiles of six novel C-type lectins in response to bacterial and 20E injection in the cotton bollworm (*Helicoverpa armigera*). Dev. Comp. Immunol. 37, 221-232.

Wang, J.L., Zhang, Q., Tang, L., Chen, L., Liu, X.S., Wang, Y.F., 2014. Involvement of a pattern recognition receptor C-type lectin 7 in enhancing cellular encapsulation and melanization

due to its carboxyl-terminal CRD domain in the cotton bollworm, *Helicoverpa armigera*. Dev. Comp. Immunol. 44, 21-29.

Watanabe, A., Miyazawa, S., Kitami, M., Tabunoki, H., Ueda, K., Sato, R., 2006. Characterization of a novel C-type lectin, *Bombyx mori* multibinding protein, from the *B. mori* hemolymph: mechanism of wide-range microorganism recognition and role in immunity. J. Immunol. 177, 4594-4604.

Xialu, W., Jinghai, Z., Ying, C., Youlei, M., Wenjun, Z., Guoyuan, D., Wei, L., Mingyi, Z., Chunfu, W., Rong, Z., 2013. A novel pattern recognition protein of the Chinese oak silkmoth, *Antheraea pernyi*, is involved in the pro-PO activating system. BMB Rep 46, 358-363.

Yu, X.-Q., Tracy, M.E., Ling, E., Scholz, F.R., Trenczek, T., 2005. A novel C-type immulectin-3 from *Manduca sexta* is translocated from hemolymph into the cytoplasm of hemocytes. Insect Biochem. Mol. Biol. 35, 285-295.

Yu, X.Q., Gan, H., Kanost, M.R., 1999. Immulectin, an inducible C-type lectin from an insect, *Manduca sexta*, stimulates activation of plasma prophenol oxidase. Insect Biochem. Mol. Biol. 29, 585-597.

Yu, X.Q., Ling, E., Tracy, M.E., Zhu, Y., 2006. Immulectin-4 from the tobacco hornworm *Manduca sexta* binds to lipopolysaccharide and lipoteichoic acid. Insect Mol. Biol. 15, 119-128.

Table S3. Features of 32 *T. xiaojinensis* CTLDPs

name	ID	aa sequence[a]	length	domain architecture[b]	CRD motifs[c]	note	GenBank accession number
CTL-S1	comp349455_c0_seq1	MHSYALLCAVFVAAVHAQFPNGRILEPPVPQQCVHRVVHERTSDGKGYFFSWRDPALKGVEEDWLGARNFCRKRCMDLVSLETSDENEFVKSRIVQGGIKYIWTSGRICDFKGCDRPDLLPTPVNGWFWTAELQKLAPATNRQQNDWSEGGGIGRPQPDNRELLQGGAPENCVAILNNFYNDGVHWHDVACHHRKPFVCEENDALLKYVRYTNPALQV*	218	SP-CTLD	QPD		KX550114
CTL-S2	comp95219_c0_seq1_CTL16	MQTWCLVAMTLLALAAMASGQSDRPGRFLSLPVPQKCATRPKEFAYQGRSYFYSGHVPALANKKVDWLDGRNICREYCMDLISIETQEENNLVFRLIQQNDVPYIWTSGRLCDFKGCESRRDLEPKNILGWFWSANREKLSATNQIPNGWGYNPWSQTGHKKQRQPDNAEFDINGTTESCLSVLNNVYKDGIAWHDVACYHEKPFICEDSEELLNYVAANNPNLRL*	226	SP-CTLD	QPD		KX550115
CTL-S3	comp73172_c0_seq1_CTL19	MIRHVTLVLAALMALACVASAQRRLALPDPRSCSNRVRHSTYRDARGVLHSYFFSWEHGATRSLEVDWLDARNICRRHCMDAVSLETPQENEFVKQRIARGNVRFIWTSGRKCNFAGCDRADLQPPNVNGWFWSGSGAKIGPTTQRNTGDWSYTGGYGQAQPDNREAAQGNDESCLSILNNFYNDGIKWHDVACHHVKPFVCEDSDELLNFVRSRNPGIRL*	220	SP-CTLD	QPD		KX550116
CTL-S4	comp120472_c0_seq1_CTL6	MNSATLLSCLVLVVVAAFAPVTGQRITTIQLDGVQYFISRMNPYSPELNYFLAYQYCRSLGLQLASFETKEKADSITTYLTNAGYNKYDFWTSGNNLGTDMYLWMSTGLPFNATFNYMRREAALAVDAAPASDSMDPLDMPQGSTAPQRTARHGTEHVMTNGCITLKAPSFHWEPQHCAEIKDFICEQTRCYYYNYGSIPVSSAQG*	206	SP-CTLD	QRT		KX550117

CTL-S5	comp184160_c0_seq1	...VVAPPPGKKEVSETDLYLLGAIEKMVYRVDFMEKRLRKVEEMLYYVMAGSNKKSDSCPNNYTRAGSNCYYFGHTEKYDWKTSNVVCKKLKGNLAEMETIEENQDVVAYIQSRSQLQGKDFWTGGLNPGLLWIWSNSARPVHTTKSNETQLVNHPLQDAPHSIHGSGRCLKLAYNPTLRSYAYYGFDCSVRQHYICELPDKGLSNDIDRLAKDIKLRENKYIPLS*	224	?-CTLD	HPL	lost N-terminus	KX550118
CTL-S6	comp285015_c0_seq1_CTL26	MLLCYAGEARSAGHQSPLGAIGKADCASCDDDSTLGVNRWTMPLLKLGEKRYYLGIFFKANWFRAAQYCRFHGMHLASISSQEENDKLEKYVRDSGFGNEHFWTSGTDLADEGSFFWMSSGRPLTFVNWNAGEPNNFRYDNGEEENCLELWNRDGKGLKWNDSPCSFETYFVCEVRTD*	178	CTLD	EPN		KX550119
IML1	isotig04615_CTL1	MLYFLLFLVVVAAGKSIETSKQVFKFYRNDYDFTPEFDCLYKFHTLDRTWADARAQCQAEGAELAAPDTANEAEYLHSLLSKRAPDVSGIFLGIHSQFAKGYFVSLRGVPLENIYHEWQHGEPNNYDNLEACVAMTRDGKLNDVKCDKEYPYICKKLASTLKIENDKCKTYDIAYEPTEKGDRCYKFHTEPKTWKEAYMICKSEGSYLAVMNSEEESNHLVKKFDKVSRNDLYGLTDKHRFIIGFHDMLHEGEYRTIFGQSLKDAGFVNWCQGQPDNYNGKQNCGAILHDGTLDDIECDTPYTFVCEREIKKES*	314	SP-2CTLD	EPN, QPD		KX550120
IML2	isotig04304_CTL2	MFKLIFLFIGAATCSSIGLPSLSFRDDYSYAESQKAFYKFHRVPRTWEVARFLCGAEGGQLASPESDEELKFFHGMLAGMPNSVFGLYIGAHRKFSPSTFVNLNGKRIDSVLRWWDHLEPNENDYTEDCVTVGRNGKLNDERCDKRFPFVCKVASESVVANTVCKNFDPAYQPYDKIPSRCYKLHTEPKTWHNAYQTCTSEQSNLVVVKSADEADYLSKLIKQNLPSKVIGSFAKDKIVVGFHDLFAEGEFRTVFGEKLDNIGFSSWAEGKPDEHQDGNDCGALNSEGKLEDVSCNKQFAFVCVREV*	307	SP-2CTLD	EPN, KPD		KX550121

IML3	isotig03303_CTL 3	MSTYHIMVFICFTCQLFLCPVDSVIGIATFFRRDYDLIHSQRSFYK VHNIPKSWNDARKQCILEGSTLAIPETAEEAEKLRDLMSKKIDTN SVAYLGIQAFTKGFFFTLDGRSINEIYHGWAPGQPNDVDNQEDC VILSSDGKLNDVNCVNKNPFICEKSQDSATWNNACETSDLAYTR VAGMAHCYKVHLEPKNWPDAYATCVAEQSYLAILNSRTESKAL INLFNTKPYRNVTQNFLRGAIHLGFHDRFTEGLFTTIRGDTLSDAG FDKWGGGQPDHSLVETCGSMFYDGELNDIGCEQRCFFICEREVG DGSSALSLRFAGLE*	316	SP-2CTLD	QPN, QPD	KX55012 2
IML4	isotig01895_CTL 4	MLKVSLLLILIYFNLICISGVNGDTSQFFRREYTLYSEVEAFYKVQ KTPKSWNDARKACILEGSTLAVPETAAEAEVYSKLIDDNLDDYR RLVFVGIHAFARGLFTTLDDTPIDEIYHGWAPGEPNDMDDNEYC VAIDRRGKLRDVPCLNKYPFICEKARDTVKWNNDCQIADLDYGR INGMANCYKLHLTPKNWTEAYATCTGESSSLAILNSEAESTALK QLYETKSDAKINSGNVDILLGFHDKFREGQFITIKGMSLVEAGFN SWSSGQPDHPTTETCGSMFPSGLLNDVNCALPSPFICEHENDLAI MPRRRI*	318	SP-2CTLD	EPN, QPD	KX55012 3
IML5	isotig01063_CTL 5	MFRFLPVLLYCLGINSAKMTNPFFRSDYTYEPSQESFYKIHFETKS WLEARALCEAEGTSLVMPESKPEIEYLVKIMNDNLPENVIAAYIG IHDMFSEGIFVTLTGRDVPIAHNYWAPGEPTNSNNTEHCVHILRS GKYNDIVCSNRYPFICKKTLESLTANPRCATSDLAYFPNYNTSSC YKLHLTPKTWTDAFATCHAEQSYLAIVNSADEAQFFKEQLADHP PASLHGDFFKDGIYLGFHDLFSEGSHVTILGTPLSQTGFAQWSPG QPDNAGQNENCGHMYRSGLLNDINCSSYKLIFFCERDI*	308	SP-2CTLD	EPT, QPD	KX55012 4

IML6	comp112889_c0_seq1_CTL7	MIIQLSLIICVLVEFGYVLAGENRIFRYDYEFSDEINSYFRLHKYPRSWVNARTRCRHEGAQLYTPANGQEADFVKLLHEDLHSDFNGIYLGLNAYFSEGDFASTEEQSVLDLYNVWNDGEPNNMNDIEDCAVMTRTGKYSDVACNQVFPFLCARPEAGLTWNSACNTYDMGYQSSPGLSSCFKIHTDPKNWTNGYATCKAEGGYLAIVNSVAEKSFLVKMFAENPAGSLRGNFWKDVLLIGFHDWFMAREYEYLDGPRVPLTKFSGGEPNDFAPGERCGAMYRNGLFDDIWCNRPNIFACERPLVAALPPGRGVTPTWRSSRNR*	325	SP-2CTLD	EPN, EPN	KX550125
IML7	isotig04171_CTL8	MMKKSIFFLLKILLIVPPFAIAEKTQWQYMGSQFREDYQLQPDLKAFYKIHRTPLTWFDARTRCDREGAELVAPQSHEEVEMLKTLLEKLDMHEPFLFLGIHSLFAKGRFVTLSDVDVQNLNLHWESGEPNNYKNNENCVTLNYNGRLSDTDCNDVHPFVCKKVARNISWNNECYTYDQEYKLDSSLSTCYKLHTQPKNWTSAYDVCESEGGHLAIINSVEEKNFLVKLLGEHPPARIQGNFNKDMTFLGFHDLFVSREFRTITGETLQEAGYNEFAGNQPDNVSPGEHCGAMFRNGQLDDLWCDRPYLFICERSNELRERPQDPFMKHFSKELVYIVPA*	339	SP-2CTLD	EPN, QPD	KX550126
IML8	isotig03362_CTL9	MLITYIFLIQLLAGTLYAATAGTKQWQYTGSQFREYYEFKSSLKAFYKVHRVPLTWHDARTRCDREGAELVAPETYEEVDMLKSVLEKVNAHNPSLFLGIHSLFMKGNFVTLSDVNLEDLSLTWEFGEPNNIANNENCITMNQMGRLADTDCNSVHPFVCKRKAKNMSWNNECNTHDAAYTLAPSLSTCYKLHTQPKNWTTGYDVCKAEGGYLAIINSIEERDFLAKLLEKHPPESILGNFGKDIIYIGFHDLFVNREYRTLEGQTLAEAGYNQFAGNQPDDAPPGERCGSIFRNAQLNDAWCDRLFLFICERSIKKRGKAESLSAQ*	327	SP-2CTLD	EPN, QPD	KX550127

IML9	comp119095_c0_seq1_CTL10	MWFLNIFLIVAVAGGTIEVSPNEFLELYPDEYTYASTLDCLYKVH<br>ALPRTWDEARFQCQAEGAELAVPVNVEEAEYLHNLLCKLETPSQ<br>GIYTGLHSLFAKGYFVSPTGVPVENLFNKWDCGEPDDSNEDQDC<br>VILGMSGKLDDKSCNRRYPYVCKKLSSSLKTNNARCQTYDLAY<br>QPVESGDRCYKFHLEPKNWKEAYAICRAEGAHLVVLNSEDEMR<br>YLTKKFDKLARNDLYGPTDKHRFLIGFNDLLHEGEFRTIFGQTLQ<br>AAGYESWCSGQPDDRNRTQNCGAILHDGTLDDVECDKQYVFAC<br>EKETNLL*	314	SP-2CTLD	EPD, QPD	KX550128
IML10	isotig04092_CTL11	MFRAFLMISCCLIAATDAAATSKDDPYFRSDYKYESSLAAFYKV<br>HKSAKYWTDARAACQYEGASLVVPETQAEINVLVQLINEQLKN<br>DTTGIFVGIHDLFIEGTFMTINGEEIDDIFENWAAGEPNDVNDNED<br>CVLLHRNSKYYDINCKSKFPFICKKTLATLTANPMCETADKAYFP<br>DAAGDKCYKLHLEPKTWSEAFTICHAEQSYLAIINNDVEAKLLK<br>ETLAKHPKDTLKGNFYKDGAYLGFHDQFTEGEFVTVFGQRLAET<br>GYVEWSPGQPDNANKNENCGSIFRSGLLNDINCAYKLLYFCERD<br>TGDANAVGLFSRFQENSPTVQ*	331	SP-2CTLD	EPN, QPD	KX550129
IML11	comp123926_c0_seq2_CTL12	MLSALFVIPCCLIATTNVVVAQDTDPNFRSDYKYQPTMAAFYKV<br>HKSPKYWTEAVSTCKYEGGYLVVPESQGEIDFLVKLIENQLSGD<br>TTGVFVGIHDMFIEGNFMTVKADSISDIFDNWAAGEPDDFQDNE<br>DCVLLHRNSKYYDYDCKRKFPFICKKTLASLTANPLCGTSDKAYI<br>PDVTGDKCYKLHLNPKTWSEAYTICHAEQSYLAVPTNKKESDLL<br>KEMLAKHPENTLNGNFYKNGAYLGFHDQFTEGEYVTVFGEPLS<br>QVSVVEWSPGQPDNGLNNDENCGHMFRDGLLNDVNCSWKLMF<br>FCERQIGDDDVIDLFSRVQNNIPELRSLEGS*	337	SP-2CTLD	EPD, QPD	KX550130
IML12	comp118822_c0_seq1_CTL13	MMFRILLILSCYLIAAITMNQESEPTFRPDYKYVDSEAAFYRLNIN<br>PMNWTEARSTCQEEGASLVVPENQSEIDILVELIKSKSLDNARGV<br>FMGIHDMFLTGTFLSINGQEMSEVFENWASDQPNRKAGKANCVF<br>LHRNSKYYDGKCKSKLPFICKKTLDTLTEQPLCETSDVAYFPDAT<br>ETRCYKLHLKPKTWSKAFATCRAEQGYLAIINDEEEANILKNKL<br>AEHPKRTLKGDFNKDHVFLGFHDRFTAGEFITVFGTSLSETGFTQ<br>WAPRRRNNRKNRQHCGGLMRNGFLDDINCSSKCMFFCERELGD<br>ANTVDIFSRFQDIEPETS*	329	SP-2CTLD	QPN, RRN	KX550131

IML13	isotig04145_CTL 14	...NVTRNIFSIQKGEPFFRSDYSYVPSQAAFYKIHKTEKIWSEALAL CQYEGAALVVPESKSEIDTLRKIIDHNLTNASIAVYTGISDVLVEG TFATTSGFDISEIFENWASDRPTTPPTSDTSDCVVMRRNMKYSDV VCSRKFPFICKKTLSTLTFNLICQTADINYFPDAAGNRCYKLHLKP KIWPDAYAVCRAEQGYLAIVNDAAEVKLLKDKFAEYPNNTIKG NFNKDQVYMGFHDRFIEGEYITVLGRPLSETGYEEWSPGQPDNA KYSEDCGTMFRSGLYNDVNCWVKLMFFCEREVGDSEVGIFVRF QEDTPVIQ*	320	?-2CTLD	RPT, QPD	lost N-terminus	KX55013 2
IML14	isotig01955	MFRVLPILLYFIGINSAKPTNPFFRTDYTYQPSQESFYKIHLVTKS WSEARVVCEAEGTFLVMPESIPEIEYLVKLMDENLPENVIAAYIGI HDMFAEGIFVTLTGRNVPTTHNYWSPGEPTNANNKEHCVHILRS GKYNDIDCSNRYPFICKKTLHSLTANPRCATSDVAYFPNDNTSSC YKLHLEPKTWSDAFVTCQAEQSYLAIINSAGEAQFFKEKLADHPP ASLNGTQNHLGVYLGFHDMFTEGSYITIQGTTLSQAGFAQWSPG QPDDVGYRQQNCGHMFRSGLLDDINCFYKLIFFCERDIENTGHFI SFRNLVSTS*	324	SP-2CTLD	EPT, QPD		KX55013 3
IML15	isotig05113_CTL 17	MRKYRLLLISAYLICRYRVNGNTTGFFRTDYTLHSEVGTFYKAH KNPKSWNEARKWCMLEGATLAVPKTGIEADIYKTIMDDKLDAF VHSVYVGIQAFSKGLFTSLDGTPIDNLYHNWRPNEPNDLGNNED CLVIDRLTNGLNDVPCDHSYPFICEKSQDSVKWNNDCQIADPDY RRLDGMQHCYKLHKKKKNWADSYATCTAEQSYLAILNSEEESN ALKQLVASESGGNVQRYEIFHLGFHDRYAEGEFKTIQGMTLHEA GFETWDSDPYQPDHPGLENCGSMYGNGKLNDITCSIPLLFICELE NNSALSTRMSG*	318	SP-2CTLD	EPN, QPD		KX55013 4
IML16	comp98872_c0_s eq1	...EQSALDLYDEWNDGEPNNQNDNEDCAVMTRTGKYSDVACDQ VFPFLCVRSKVGLTWNSACNTYDTDYQSSAGLSSCYKIHTNPKN WTDAYGTCKAEGGYLAIVKTDAEKTFLIKLFADHPSSSLRGEFW KDALLIGFHDLFVSREYEYIDGPLVPLIKFSGAEPDNYTPGERCGT MYRDGLFDDIWCDRPNIFACEMPAVARTAKNVTPLRFRKQ*	215	?-2CTLD	EPN, EPD	lost N-terminus	KX55013 5

IML17	isotig01874_CTL20	METFSIFTILVCLLSYFRSTSCAAATFFRRDYDFFCDQGAFYKVHTVPKTWNDARKQCILEGSALAVPNDGAEAEVFRKIMEVKLGAKAVAFLGIQAFSKGIFTTLDGIFIDDIYHNWAPGEPNDAGGTEDCIIMDQLGKLNDENCERKYAFICEKSQDSVMWNDACETSDLEYTRVDNMEHCYKVHLEPKNWTDAYAICVHEQSYLAILNSREESKALIKLFQTKPHRDVRGNFLSGAIHLGFHDRFKDGVFTTIQGQPLAEAGFEKWGGGQPDGPGGETCGSMFYDGELNDVGCNVRCFFICEREAGDGGSALSVRFG...	320	SP-2CTLD-?	EPN, QPD	lost C-terminus	KX550136
IML18	comp148184_c0_seq1	...RCYKINTEAKTWKEAYQICTAEGSYLAVLNSEKEMKYLTRKFSDIYNDKIYGNFFKNVMLIGFHDLLHEGEYRTIFGEKLEEAGFENWCHGQPDNDKNDQHCGGMLRDGTLDDVSCDKQFIFACEREIKSPVKSINSTTQ*	140	?-CTLD	QPD	lost N-terminus	KX550137
IML19	comp81800_c0_seq1_CTL23	MSSFKSVCFFLLFMCVLVTISGSYIERLKLDPPPHNKGYDYTKTLNAFYKVHDLPQTWNTARAICEADGAALMWFHNKDEAVEMLVNYPKHYKQIFLGIHSKFSKRVFVTVKGLQIEDVYNNWAIGQPDNVEDDVDCVSISPEGLTAVVDCNMRLPFMCRRDYDGIINYLNNDCNTYDKGYIYKEQMKRCYKVHTEPKNWTDAYAICFAEGSYLAVINSLEESKILVKLINHVHVYGNYWFDKTQYLLGFHDMFAEGEYRTIFGETLKEAGFDQFASGQPNNVDEQNCGGMTTEGNLNDLWCTKPFPFICARDVSLKPW...	319	SP-2CTLD-?	QPD, QPN	lost C-terminus	KX550138
IML20	comp99460_c0_seq4_CTL24	...SAAATFFRRDYEFYKNQRAFYKVHAIPKCLNQARIQCNLEASTLAVPKTAAEAEIFKDLMKKTINPNSVAYLGILALSKGIFTTFDGIPIDDMYHEWAPGQPDDANDEYCVVMGSDGKLSDINCFYKNPFICEKSQDSVTWNYACETSDLAYTRVPEMDKCYKVHLEPKNWAEAYATCAAEQSYLAILNSKEESTSLINLFNTTPHRYVSGNYAIGAIHLGYYDGFAQGVFRTLQGQTLTNAGFDKWAYMQPDHPRSEYCGSMFYNGELNDIGCEYRCFFICEREAGDGSSALSIRNANVY*	310	?-2CTLD	QPD, QPD	lost N-terminus	KX550139

IML21	comp102009_c0_seq2	MYKYRLLLVLAYLIWQYRVNGSTTVFFRRDYTLHSKVGIFYKAH KNPKSWNEARKICILEGATLAVPKTAIEAEVYKQIMDDKLDSFV HSVYVGIQAFSKGLFTSLDGTPIENIYHNWRPNEPNDLDNNEDCV IIDRLTGLNDVACYHRHPFICEKSQDSVKWNNDCQIADPDYRRL DGMQHCYKL…	186	SP-CTLD-?	EPN	lost C-terminus	KX550140
IML22	isotig03477	MFRVALILLPSWYIISINASAIQKADPYFRSDYDYVASQAAFYKIH KTPRTWTDALALCQYEGAMLIVPESKDEIYILKEIIDNTLTNASIA VYTGINDIIMEGIFLTITGIDISDIFENWAPDRPNDDGAQSDCVAM RRNMEYSDVACGRKFPFVCKKTLATLTVNEICQTFDKNYFPDAT ASRCYKLHLAAKNWSQAYKVCRAEQAYLAILNSAGEANFLKDK LTVYPKDSLKGNFGREEVFMGFHDKFVEGEFMTVFGQPLAETGF EEWSSGQPDDARSNEDCGTLSRSGLLNDIDCFAKRMFFCERELE DSATTARVI*	322	SP-2CTLD	RPN, QPD		KX550141
IML23	isotig04483_CTL30	MFRFLPIVLYCLGINSAKVIIPFFRSDYAYEPSQESFYKMHLVTKS WTEARAVCEAEGTSLVMPESKSEIEYLVKLMDENFPKHMIAAHI GIHDIFAEGIYVTLAGHYVSTTGDYWSPDEPNNFNNKEDCVHIRR SGKYNDIDCSSIYPFICKKTLDSLTENPRCATSDLAYFPGDNTGSC YKLHLTPETWSDAFATCYAEQSYLAIINSADEAQLFKQKMADYP PDSLHGEFFKDGIYLGFHDMFSEGRHVTIQGTLLSQTGFAQWSPG QPDNNSENENCGHMYRSGLLNDINCSCKLIFFCERDIENSERNNIS IKNLVTSN*	324	SP-2CTLD	EPN, QPD		KX550142

CTL-X2	comp99293_c0_seq4_CTL25	...GTNVAFRKPANQSTTVRGGGAANGNDGEKTTEHDGKRCTETQ<br>REASPWWQVDLLRHYAVKVVRVTTRGCCGHQPLQDLEIRVGNS<br>STDLQRNPLCAWFPGTIDEGVTKTFTCARPLIGQHVFLQLVGVEG<br>SLSLCEVEIFTTEEFSNDRCAPPSAPADIQLAAFSRTCYEFNVAKG<br>GSFEEARKYCQGHGGDLIHGFQGATSSFLLQELERRKAQLKTQL<br>VWVGAEKEPGLTSRTWRWVNGETVSRPAWGKDQPNNYNGEQN<br>CAVLDGGRSWLWNDVGCNLDYLHWICQHLPAACGSPDKLLNT<br>TIIGHNYTVGSEITYKCPVGHMLIGAAARTCGKDGFWTATAPSC<br>KYVDCEGLSPMADGLVTLIDNRTTHGAVALYTCHENYTLIGKSE<br>RVCSDDGTWSESVPQCLFDWCPDPPNVVGSTVEVDGHKAGSLA<br>TYTCHPGFILFGQQTVTCTLGGTWTGTPPSCKYIDCGTPAQIDRG<br>SFNLLNGSTTYGSYAQYSCEADHWLDGAEYLACNRDGKWSHDT<br>PACELITCDEPEVPPGGYVVGYDLNVHSSIEYHCEVGHKLVGEST<br>LTCQQDGEWSREAPLCEYVDCGKLPSMLYGSAEYINGTTHLASL<br>IHYSCTTNYRLVGPVTRICQDNHQWSDTSPKCEEIRCPEPIVADNS<br>IVSVTGNDRMYGRTLIRTADSYSSNAMTYKIGALVKYRCERGYK<br>VIGESLSTCEDTGAWSGNTPKCQYVDCGNPGRIANGKVTLATNA<br>TYYGAAALYECDKNWQLDGVSRRLCQDNGTWSSEAPVCKEITC<br>NDPSTFQRELSPGLHVITSTRSIGGVAHYRCDRGYTLQGNTTRTC<br>QPRGSWNGQQPICLPVDCRDPGIIENGRIIITNGTTLFASTIEYHCL<br>PHYQRIGPFLRKCMEDGHWSGEEPSCELISNETQDSSNLPLSVGIG<br>CGVVLFLLMLLGIIYMRLRKATPVKNTENVEGAERKEDQNAAV<br>MSYSTLHDGNNGIRGNHIYDHVNDNVYDSPYSDHLTDNHTYNR<br>SQGDDSDTTYEPEPQNGPSAVVTINGVAVR*	1043	?-FTP-CTLD-10CCP-TM	QPN	lost N-terminus	KX550143

CTL-X4	comp98113_c0_seq1	MKSVLSILFLTVCLSNSQVYQQVPEGTDPYGQNPTYGQAPTYGQ<br>GSSYPNYQQPNLQSNYLNQSNLGYNNYGANYPQQNEYQNNYN<br>QQDPFNSGNYRADDFINPSSPNDVDSSSYYGNRDLDFSFRPQCPQ<br>NWILFQASCYRFMKSPLRPRNEARKICQAYESDLISINSVEEHGFII<br>NELLKLDPQHRKWYISARQQSPQYWTNDGDGTQMSNMENAFLT<br>DNHMVYQQDQIGRDYLVYKFSDISRKWGFEQVDGYEPLLFICEA<br>PVNKLKVLVDDDRSFTYGLNIDDPNKIPRGPYFIRQPEDKTFDLS<br>KRTIMNEVQLKCLAGGYPAPTYAWYKEEYEFDKLVPKKIDPLSD<br>SRYTVSGGTLIIYNPENIIDGSNYHCVATNKYGSIKSESVLLSFGYI<br>REFNLKRSSEQGNQYWGKVLYCDPPPFYPSVNYYWSRDYFPNF<br>VEEDERVFVSNDGGLYFTALEPIDRGIYSCNVKSRVSDTGRNGPF<br>FNLYVYPHADFQQLKFPNNFPKAFPDAPVVSHEVRLECMAFGYP<br>VPSYNWTRTGGRIPRHAQMSSFGRILTIPSVSIEDEGEYVCTITND<br>RASINNSVVLKIQAYPNFTIPLTDKHMDNKGDLTWTCEAFGVPD<br>VNYTWWKNGERLTMDTIAPEDRDRYHIQDNILTIKYLDPTKDPG<br>MYQCAARNQLKTTYSSAQLRVLSLKPSFKKRPLESETYGAEGGN<br>VTIACNPEAAPKPTFTWKKDNNVIGGGGKRRILENGNLVISPVSR<br>DDEGVYMCAAKNQYGNDESLGRLIVLRAPRFIEPLPTRINTLFGQ<br>SLFLHCNAETDEILDTAFIWNHNGMRIKEVGDYFADKRL...	840	SP-CTLD-5Ig-?	DNH	lost C-terminus	KX550144

CTL-X6	comp77692_c0_seq1	...SEESSFTPLKDLNLPYNLFSMIDICDTSKVVTVEERVLVYYKYDNNPVNCVKIFRSVFRVKPFGFRLLQFHLFNHTRTYGKRDALSLYDGDIFNVTAPMIGYLEHGSPDEKKLFRTEGARLSVKLFANGAPSFHGFIAEVVTLPISAIGFNRDVQHNISNSEFIENRQGAISYHSVGEVNPVLTMDRNQFMRNCKQLYGNFTTCQSALRVDVQNTQTLYFRNNLVSENQGGLYLRADSRGSATSLKGWLHNNLFTANLNLPCLRIEGRQSSPYQEVTIYRNYFTRNWTPFKDVIVLRQVVSNFTHNYVHYNTGLRILQVSGFDKVRLPIYQTTSHNGFYSNHALDRDGRATVVAGTAGQHYVDNIFFNPDNDYEMITVNRSVQLDLWRTRIDAAHNYWSYNESYAVGGRIRDKSDNPLLLEVEYRPYYMNNASVLNGKCPPGWDLVGGTCYMYVGAPMNYEDARRFCLSDNASMPYISGTSYFELYRFLQRQNQWFQFNDRVWVQHIDFVTQCTSFSFQTIQVDDCQRKSAFLCEIDPKIFIDPMFWRDDAVIVGVVGVLAGTILLVAAAIACWYSKSRHRHAQRLERRNSIRQSLHSVRSIGSMNGGFADISYRRKMGQLSTRSTDTLTKNSDYKKMLASSASVDSVEKSQFNSSLEDNQSYDIYEAHNPNPNNIPLKHSSFNKRTSPPEYSAPINRASYDLAFRNEGFRDISTNPSVGQSANTVLTEEIPIIHHPGGIASTSQESAYGDDDTLSPNSDNQYYNADTLPLRGNTMGSSSDTLDFKRGLEKETNGYNQYGAPVMYGKQPKLSFLKELRHRSLNADQPPPNVAMPQTTFGQRRNNIVDQQQYFDDNLPSPPHPPGYESYDTSPDQHLPGYDSTPSQNYSNDFSHSHGMPKSRSKSEALLETSFDFDDSDDSTAGPLPMTEAHRSMSQPLETAM*	942	?-CUB-2PbH1-CTLD-TM	?	lost N-terminus	KX550145

a : aa sequence means amino acid sequence. The signal peptide in each sequence is shaded in blue.

b: The missing parts in the CTLDPs are indicated by "?".

c : The Glu-Pro-Asn (EPN) and Gln-Pro-Asp (QPD) motifs are highlighted in blue and orange, respectively. The CRD motif in CTL-X6 could not be idenfied and uesd "?" to deno

Table S4. Primers for 6 genes encoding CTLDPs

Oligonucleotide Name	Sequence
IML1-F	TTCGTGAACTGGTGTCAAGG
IML1-R	TGTCGCACTCAATGTCATCC
IML2-F	AGCGTTTCCCGTTCATAT
IML2-R	TAAGCATTGTGCCAGGTC
IML8-F	CTGTGCTGGAGAAGGTCAATG
IML8-R	GTTCGCAATGTTGTTCGGTTC
IML10-F	CAGCAGGCGACAAGTGTTAT
IML10-R	TGATAGCGAGGTACGACTGTT
IML11-F	TGGCTAAGCACCCTGAAA
IML11-R	CAACACTGACCTGCGAAA
IML17-F	GTGATGTGGAATGATGCTTGTG
IML17-R	CTAGATGGATTGCTCCGCTTAG
rpS3q-F	GAGAATTGGCTGAAGATGGT
rpS3q-R	GAGGTCAACTCCCTGATACG

Table S5. Structural characteristics of 53 CTLDs in *T. xiaojinensis*

Domain	Motif	Sugar[a]	Binding site residues	C-score[b]	Ca^{2+}	Binding site residues	C-score[b]
CTL-S1	QPD	FUC	125,127,129,**138**,155,156,157	0.59	2	[76,80,128,**138**,139] [48,50,54,168]	0.15; 0.12
CTL-S2	QPD	MAN	129,131,133,**142**,159,160,161	0.64	2	[47,49,53,172] [75,79,132,**142**,143]	0.09; 0.07
CTL-S3	QPD	TRE	73,129,131,133,**141**,147,158,159,164	0.63	2	[52,54,58,171] [80,84,132,**141**,142]	0.11; 0.09
CTL-S4	-	TRE	63,94,96,98,135,141,148,149,154	0.30	0	-	-
CTL-S5	APH	MAN	102,104,106,**110**,127,128	0.36	2	[39,41,45,140] [70,72,105,**110**,111]	0.20; 0.14
CTL-S6	EPN	NGA	76,108,110,112,**120**,124,126,136,137	0.82	2	[83,97,111,**120**,121] [53,55,59,149]	0.25; 0.13
IML1A	EPN	MAN	97,**99**,**105**,117,118,120	0.91	2	[71,75,**99**,**105**,106] [41,43,47,130]	0.16; 0.15
IML2A	EPN	MAN	97,**99**,**105**,117,118,120	0.91	2	[71,75,**99**,**105**,106] [41,43,47,130]	0.16; 0.15
IML3A	QPN	NGA	63,96,98,100,**104**,108,110,116,117	0.90	1	[70,74,77,99,**104**,105]	0.20
IML4A	EPN	TRE	65,97,99,101,105,111,117,118,123	0.72	1	[41,43,47,130]	0.14
IML5A	EPT	TRE	65,98,100,102,**106**,112,118,119,124	0.86	2	[72,76,101,**106**,107] [41,43,47,131]	0.40; 0.12
IML6A	EPN	GQ2	64,97,99,101,109,117,118,119,125	0.85	2	[71,75,100,105,106] [41,43,47,130]	0.30; 0.08
IML7A	EPN	MAN	63,97,99,**105**,117,118,120	0.86	2	[71,75,100,**105**,106] [41,43,47,130]	0.25; 0.12
IML8A	EPN	NGA	64,97,99,101,**105**,109,111,117,118	0.86	2	[71,75,100,**105**,106] [41,43,47,130]	0.22; 0.13
IML9A	EPD	MMA	97,99,101,**105**,117,118,119	0.74	2	[71,75,100,**105**,106] [41,43,47,130]	0.19; 0.11
IML10A	EPN	TRE	65,98,100,102,**106**,112,118,119,124	0.82	2	[72,76,101,**106**,107] [41,43,47,131]	0.52; 0.12
IML11A	EPD	TRE	65,98,100,102,**106**,112,118,119,124	0.79	2	[72,76,101,**106**,107] [41,43,47,131]	0.52; 0.13
IML12A	QPN	GQ2	65,98,100,101,110,118,119,120,126	0.79	1	[41,43,47,131]	0.14
IML13A	RPT	NGA	98,100,102,**108**,120,121,122	0.68	2	[72,76,101,**108**,109] [41,43,47,133]	0.45; 0.14
IML14A	EPT	TRE	65,98,100,102,**106**,112,118,119,124	0.86	2	[72,76,101,**106**,107] [41,43,47,131]	0.38; 0.12
IML15A	EPN	MAN	97,99,**105**,118,119,121	0.91	2	[71,75,100,**105**,106] [41,43,47,131]	0.20; 0.14
IML16A	EPN	FUC	15,17,19,**23**,35,36,37	0.98	1	[18,21,22,**23**,24]	0.13
IML17A	EPN	MAN	96,98,**104**,116,117,119	0.90	2	[70,74,99,**104**,105] [41,43,47,129]	0.15; 0.14
IML19A	QPD	MBG	94,96,98,**102**,114,115	0.59	2	[68,72,97,**102**,103] [41,43,47,127]	0.31; 0.12
IML20A	QPD	NGA	96,98,100,**103**,115,**116**,117	0.67	2	[71,74,77,**103**,**116**][41,43,47,128]	0.22; 0.17
IML21	EPN	MAN	97,99,**105**,117,118,120	0.92	2	[71,75,78,100,**105**,106] [41,43,47,130]	0.22; 0.14
IML22A	RPN	NGA	65,98,100,102,**106**,110,112,118,119	0.78	2	[72,76,101,**106**,107] [41,43,47,131]	0.31; 0.13
IML23A	EPN	TRE	65,98,100,102,**106**,112,118,119,124	0.87	2	[72,76,101,**106**,107] [41,43,47,131]	0.48; 0.12
IML1B	QPD	NGA	73,107,109,111,**115**,119,121,127,128	0.52	2	[80,84,110,**115**,116] [43,45,49,140]	0.15; 0.13
IML2B	KPD	NGA	74,108,110,112,**116**,120,122,128,129	0.71	2	[81,85,111,**116**,117] [44,46,50,141]	0.42; 0.08
IML3B	QPD	NGA	73,107,109,111,**114**,118,120,126,127	0.81	2	[80,84,110,**114**,115] [43,45,49,139]	0.27; 0.12
IML4B	QPD	GQ2	62,105,107,**108**,116,124,125,126,132	0.86	2	[78,82,**108**,112,113] [43,45,49,137]	0.28,0.14
IML5B	QPD	NGA	73,107,109,111,**115**,119,121,127,128	0.80	2	[80,84,110,**115**,116] [43,45,49]	0.35; 0.06
IML6B	EPN	GQ2	73,104,106,**107**,116,124,125,126,132	0.81	2	[80,84,**107**,112,113] [43,45,49,137]	0.19; 0.13
IML7B	QPD	GQ2	62,107,109,**110**,119,127,128,129,135	0.85	2	[43,45,49,140] [80,84,**110**,115,116]	0.15; 0.14
IML8B	QPD	GQ2	62,107,109,**110**,119,127,128,129,135	0.86	2	[43,45,49,140] [80,84,**110**,115,116]	0.14; 0.14
IML9B	QPD	TRE	73,107,109,111,**115**,121,127,128,133	0.8	2	[80,84,**115**,116] [43,45,49,140]	0.24; 0.15
IML10B	QPD	GQ2	73,107,109,**110**,119,127,128,129,135	0.83	2	[80,84,**110**,115,116] [43,45,49,140]	0.30; 0.12
IML11B	QPD	NGA	73,107,109,111,**116**,120,122,128,129	0.77	2	[80,84,110,**116**,117] [43,45,49,141]	0.34; 0.12
IML12B	RRR	GQ2	73,107,109,110,119,127,128,129,135	0.73	1	[43,45,49,140]	0.14
IML13B	QPD	TRE	73,107,109,111,**115**,121,127,128,133	0.78	2	[80,84,110,**115**,116] [43,45,49,140]	0.46; 0.11
IML14B	QPD	GQ2	62,107,109,**110**,120,128,129,130,136	0.83	2	[80,84,**110**,116,117] [43,45,49,141]	0.25; 0.15
IML15B	QPD	GQ2	62,106,108,109,117,125,126,127,133	0.81	1	[43,45,49,138]	0.13
IML16B	EPD	NGA	73,104,106,108,**112**,116,118,124,125	0.79	2	[80,84,107,**112**,113] [43,45,49,137]	0.21; 0.14
IML17B	QPD	GQ2	73,107,**109**,110,118,126,127,128,134	0.82	2	[80,84,**109**,114,115] [43,45,49,139]	0.20; 0.16
IML18	QPN	NGA	58,92,94,96,**100**,104,106,112,113	0.80	1	[65,69,95,**100**,101]	0.32
IML19B	QPD	GQ2	72,106,108,109,117,125,126,127,133	0.82	2	[79,83,114] [43,45,49,138]	0.30; 0.10
IML20B	QPD	4RS	73,107,109,**114**,120,126,**127**,132	0.82	2	[80,84,87,**114**,**127**][43,45,49,139]	0.34; 0.14
IML22B	QPD	NGA	73,107,109,111,**115**,119,121,127,128	0.78	2	[80,84,110,**115**,116] [43,45,49,140]	0.41; 0.12
IML23B	QPD	GQ2	62,107,109,110,119,127,128,129,135	0.84	1	[43,45,49,140]	0.11
CTL-X2	QPN	NGA	71,104,106,108,112,116,118,126,127	0.65	1	[46,48,52,140]	0.04
CTL-X4	DNH	MAN	94,96,98,123,124,125	0.22	1	[39,41,45,136]	0.30
CTL-X6	?	-	-	-	1	[38,40,44,97]	0.18

[a] : FUC, alpha-L-fucopyranose; MAN, alpha-D-mannose; TRE, trehalose; NGA, N-acetyl-D-galactosamine; GAL, beta-D-galactose; GQ2, a viral ligand of human herpesvirus 6 (HHV-6); MMA, methyl alpha-D-mannopyranoside; MBG, methyl-beta-galactose; 4RS, trehalose mono-butyrate.

[b]: C-score is the confidence score of the prediction. It is calculated through the COACH (http://zhanglab.ccmb.med.umich.edu/COACH/) and I-TASSER (http://zhanglab.ccmb.med.umich.edu/I-TASSER/) server. Value is in the range [0 - 1], wherein a higher value suggests a more reliable prediction.

Table S6. FPKM values of 32 *T. xiaojinensis* genes encoding CTLDP in ten libraries

geneID	name	CK	RS	Os12	Os48	Os72	Os1y	Cm12	Cm48	Cm72	Ec
isotig04615_CTL1	IML1	1.72	116.44	341.16	381.78	399.05	0.41	298.25	5768.7	5742.38	610.62
isotig04304_CTL2	IML2	6.96	96.39	523.09	85.74	101.93	4.89	684.03	4994.61	1007.56	542.48
isotig03303_CTL3	IML3	180.34	207.73	235.76	241.97	434.79	158.37	231.82	340.88	375.58	193.35
isotig01895_CTL4	IML4	527.37	1016.76	1193.28	946.94	1137.1	449.9	1502.21	1630.66	2813.89	928.42
isotig01063_CTL5	IML5	10.51	73.59	97.55	89.41	174.7	9.91	94.3	233.03	222.74	64.89
comp112889_c0_seq1_CTL7	IML6	9.24	20.58	20.65	22.03	44.41	6.64	15.84	21.13	35.28	17.47
isotig04171_CTL8	IML7	1124.16	606.27	308.4	259.69	174.74	984.79	746.4	177.4	49.85	1239.33
isotig03362_CTL9	IML8	1267.23	1272.77	1553.98	1522.19	998.01	1276.53	1394.59	1608.12	3560.94	1354.96
comp119095_c0_seq1_CTL1	IML9	1664.14	844.83	643	1390.3	893.5	1542.36	1000.97	737.65	519.7	1269.69
isotig04092_CTL11	IML10	44.42	752.76	763.96	236.02	424.53	42.79	1050.8	992.51	584.81	709.13
comp123926_c0_seq2_CTL1	IML11	0.04	2.18	6.75	2.88	20.64	0.22	15.35	15.07	9.95	9.87
comp118822_c0_seq1_CTL1	IML12	0.75	7.1	6.89	4.52	13.97	0.74	10.2	11.83	11.05	13.53
isotig04145_CTL14	IML13	104.92	237.84	193.92	268.07	383.33	88.89	193.73	158.04	218.33	210.43
isotig01955	IML14	652.18	923.07	900.47	847.22	1360.04	745.96	799.8	1096.28	1069.62	616.97
isotig05113_CTL17	IML15	0.51	3.48	13.89	36	10.58	0.24	2.61	2.98	80.76	33.01
comp98872_c0_seq1	IML16	1.08	2.14	12.93	5.77	0.29	1.25	0.93	1.55	4.68	2.36
isotig01874_CTL20	IML17	60.69	124.28	145.11	95.13	113.02	64.47	257.27	202.72	202.14	63.69
comp148184_c0_seq1	IML18	0.13	0.25	0.54	0	0.38	0.63	1.72	0.26	0.41	0.73
comp81800_c0_seq1_CTL23	IML19	0	0.12	0	0.07	0	0	0.12	0.25	0	3.15
comp99460_c0_seq4_CTL24	IML20	17.93	12.11	16.91	32.61	41.05	18.1	16.04	24.46	43.34	24.02
comp102009_c0_seq2	IML21	0.09	2.45	0.73	56.01	3.89	0	2.48	2.57	7.49	0.47
isotig03477	IML22	2.16	18.28	26.85	31.62	65.15	2.23	48.22	224.54	9.24	5.71
isotig04483_CTL30	IML23	151.96	295.51	210.39	114.89	229.47	151.08	258.58	307.94	412.14	164.05
comp349455_c0_seq1	CTL-S1	0.29	0.29	0.32	0.5	0	0	0.24	0.15	0.08	0.53
comp95219_c0_seq1_CTL16	CTL-S2	4.67	3.42	3.21	5.91	1.08	4.75	5.1	1.79	1.14	6.63
comp73172_c0_seq1_CTL19	CTL-S3	0.44	0.29	0.51	3.8	0	0.55	0.57	0	0.47	0.18
comp120472_c0_seq1_CTL6	CTL-S4	0.73	0.9	0.68	2.04	0.44	0.21	1.22	0.93	2.17	2.92
comp184160_c0_seq1	CTL-S5	0	0	1.45	4.24	0	0	0.73	0	3.13	0.06
comp285015_c0_seq1_CTL2	CTL-S6	0.27	0.18	0.71	1.69	0	0.08	0.17	0.07	1.88	0.59
comp99293_c0_seq4_CTL25	CTL-X2	0.45	0.67	1.04	2.15	2.02	0.39	0.8	0.53	2.73	1.55
comp98113_c0_seq1	CTL-X4	7.72	6.44	6.21	7.11	5.48	7.68	7.48	10.2	9.74	5.11

comp77692_c0_seq1	CTL-X6	0.41	0.44	0.55	1.29	0.34	0.12	0.42	1.08	0.35	0.8
